# CCL5 is essential for axonogenesis and neuronal restoration after brain injury

**DOI:** 10.1186/s12929-024-01083-w

**Published:** 2024-09-17

**Authors:** Man-Hau Ho, Yih-Jeng Tsai, Chia-Yen Chen, Anastasia Yang, Thierry Burnouf, Yun Wang, Yung-Hsiao Chiang, Barry J. Hoffer, Szu-Yi Chou

**Affiliations:** 1 Program in Medical Neuroscience, College of Medical Science and Technology, Taipei Medical University and National Health Research Institute, Taipei, 11031 Taiwan; 2 Graduate Institute of Neural Regenerative Medicine, College of Medical Science and Technology, Taipei Medical University, 250 Wu-Xing Street, Taipei City, 11031 Taiwan; 3 Department of Otolaryngology Head and Neck Surgery, Shin Kong Wu Ho-Su Memorial Hospital, Taipei, 11160 Taiwan; 4 School of Medicine, Fu Jen Catholic University, New Taipei City, 24352 Taiwan; 5 Department of Molecular and Cell Biology, University of California, Berkeley, LA 94720 USA; 6 Neuroscience Research Center, Taipei Medical University, Taipei, 11031 Taiwan; 7 International PhD Program in Biomedical Engineering, College of Biomedical Engineering, Taipei Medical University, Taipei, 11031 Taiwan; 8 Graduate Institute of Biomedical Materials and Tissue Engineering, College of Biomedical Engineering, Taipei Medical University, Taipei, 11031 Taiwan; 9 NeuroTMULille International Laboratory, Taipei Medical University, Taipei, 11031 Taiwan; 10 International PhD Program in Cell Therapy and Regeneration Medicine, College of Medicine, Taipei Medical University, Taipei, 11031 Taiwan; 11 Center for Neuropsychiatric Research, National Health Research Institutes, Miaoli County, Zhunan, 350401 Taiwan; 12 Department of Neurosurgery, Taipei Medical University Hospital, Taipei, 11031 Taiwan; 13 Department of Surgery, School of Medicine, College of Medicine, Taipei Medical University, 11031 Taipei, Taiwan; 14 Department of Neurosurgery, University Hospitals of Cleveland, Case Western Reserve University School of Medicine, Cleveland, OH 44106 USA; 15 Scientist Emeritus, National Institutes of Health, Maryland, 20892 USA; 16 International Master Program in Medical Neuroscience, College of Medical Science and Technology, Taipei Medical University, Taipei, 11031 Taiwan

**Keywords:** CCL5, Traumatic brain injury, Axon injury, Axonogenesis, Myelination

## Abstract

**Background:**

Traumatic brain injury (TBI) causes axon tearing and synapse degradation, resulting in multiple neurological dysfunctions and exacerbation of early neurodegeneration; the repair of axonal and synaptic structures is critical for restoring neuronal function. C-C Motif Chemokine Ligand 5 (CCL5) shows many neuroprotective activities.

**Method:**

A close-head weight-drop system was used to induce mild brain trauma in C57BL/6 (wild-type, WT) and CCL5 knockout (CCL5-KO) mice. The mNSS score, rotarod, beam walking, and sticker removal tests were used to assay neurological function after mTBI in different groups of mice. The restoration of motor and sensory functions was impaired in CCL5-KO mice after one month of injury, with swelling of axons and synapses from Golgi staining and reduced synaptic proteins-synaptophysin and PSD95. Administration of recombinant CCL5 (Pre-treatment: 300 pg/g once before injury; or post-treatment: 30 pg/g every 2 days, since 3 days after injury for 1 month) through intranasal delivery into mouse brain improved the motor and sensory neurological dysfunctions in CCL5-KO TBI mice.

**Results:**

Proteomic analysis using LC-MS/MS identified that the “Nervous system development and function”-related proteins, including axonogenesis, synaptogenesis, and myelination signaling pathways, were reduced in injured cortex of CCL5-KO mice; both pre-treatment and post-treatment with CCL5 augmented those pathways. Immunostaining and western blot analysis confirmed axonogenesis and synaptogenesis related Semaphorin, Ephrin, p70S6/mTOR signaling, and myelination-related Neuregulin/ErbB and FGF/FAK signaling pathways were up-regulated in the cortical tissue by CCL5 after brain injury. We also noticed cortex redevelopment after long-term administration of CCL5 after brain injury with increased Reelin positive Cajal-Rerzius Cells and CXCR4 expression. CCL5 enhanced the growth of cone filopodia in a primary neuron culture system; blocking CCL5’s receptor CCR5 by Maraviroc reduced the intensity of filopodia in growth cone and also CCL5 mediated mTOR and Rho signalling activation. Inhibiting mTOR and Rho signaling abolished CCL5 induced growth cone formation.

**Conclusions:**

CCL5 plays a critical role in starting the intrinsic neuronal regeneration system following TBI, which includes growth cone formation, axonogenesis and synaptogensis, remyelination, and the subsequent proper wiring of cortical circuits. Our study underscores the potential of CCL5 as a robust therapeutic stratagem in treating axonal injury and degeneration during the chronic phase after mild brain injury.

**Graphical Abstract:**

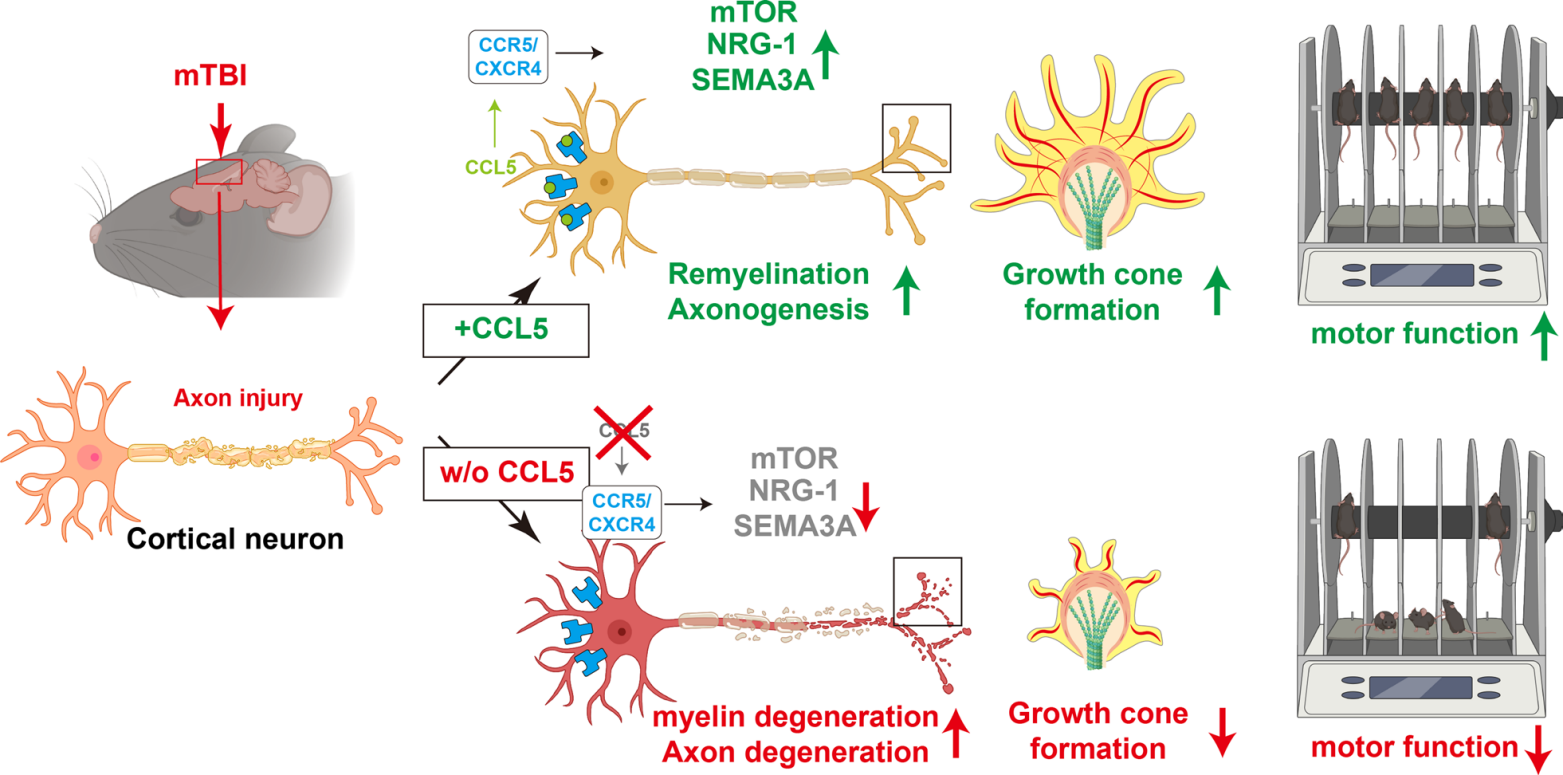

**Supplementary Information:**

The online version contains supplementary material available at 10.1186/s12929-024-01083-w.

## Introduction

Traumatic brain injury (TBI) is a complex disorder caused by external forces and is also the most significant cause of death and disability for people under the age of 40 [23, 31]. According to the Glasgow Coma Scale (GCS), TBI can be clinically divided into mild (GCS:14–15), moderate (GCS:9–13) and severe (GCS:3–8). Mild TBI is the most common brain injury caused by contact sports (i.e., hockey, football), motor vehicle accidents, and falls. Evidence from long-term studies on soldiers and athletes suggest that mild and repeated mild brain injuries are associated with the development of Chronic Traumatic Encephalopathy (CTE) [19, 37, 44], and also increase the risk of early onset Parkinsonism [18], dementia, and Alzheimer’s disease (AD) [25, 48].

Axonal shredding and tearing upon the impact, also called traumatic axonal injury (TAI) or diffuse axonal idiopathic injury (DAI), are the initial direct damages from mechanical impact on brain tissue. TAI is characterized by impaired axoplasmic transport, axonal swelling, disconnection with small hemorrhagic and/or non-hemorrhagic lesions, and brain swelling [7]. Unfortunately, much evidence suggests that neurons within the adult mammalian CNS cannot regenerate their axons after injury [13, 43]. Several deleterious cascades will be activated, leading to axon degeneration after injury. First, damaged glial cells/oligodendrocytes may not be able to provide sufficient energy to support neurons and cause neuron degeneration after injury [33, 49]. Second, increased calcium (Ca^2+^) influx leads to neuron apoptosis and degeneration [33, 49]. Third, glia scar and myelin-associated inhibitory proteins, such as Nogo, myelin-associated glycoprotein (Mag), and oligodendrocyte myelin glycoprotein (OMgp), can inhibit axonal regeneration after injury [15].

Recent evidence suggests that CNS neurons can revert to an embryonic-like growth state in the chronic/remodeling phase of injury to facilitate axon regeneration [46]. This “redevelopment” state provides a permissive microenvironment and the intracellular machinery for axon regrowth [9]. The endogenous repair systems activate and repair damaged axons within the chronic phase after axonal injury. The molecular machinery underlying axon regeneration is very similar to axon growth, such as Rho-GTPases Cdc42, Rac-1, and RhoA, phosphoinositide 3-kinase (PI3K)/AKT signaling pathways, and mitogen-activated protein kinase (MAPK) signaling [38]. Importantly, Rho is also the target molecule for myelin-associated inhibitory proteins [15]. PI3K/Akt activates its downstream molecules - the mammalian target of rapamycin (mTOR) and promotes axonal regeneration, synaptic plasticity, and neuronal survival after injury [35, 45]. Modulating the mTOR pathway is a novel strategy to promote axon regeneration [10]. Among three types of MAP kinases, the Erk cascade is a major pro-survival intracellular signaling pathway that can be activated by GDNF (glial cell-line derived neurotrophic factor) to promote neurite outgrowth in the spinal cord [29].

Chemokine CCL5; C-C motif ligand 5, also known as RANTES (*r*egulated on *a*ctivation, *n*ormal *T* cell *e*xpressed and *s*ecreted), shows many protective roles after neuronal damage, such as in stroke [51] and AD [24, 28, 32, 53]. In brain trauma, the plasma level of CCL5 increases in both human patients and animals after injury [2, 20]. Interestingly, CCL5 was found to be raised around the axonal transection site in mice immediately after injury [3]. The increased CCL5 around the axon transection site induces leukocyte infiltration to the injury site, but its function on neurons is still unclear. Our previous study identified that CCL5 is an essential factor in activating glutathione peroxidase 1 (GPX1) after brain injury, consequently reducing oxidative stress and protecting hippocampal neurons from oxidative stress-induced death; this effect facilitated memory-cognition recovery in mice after mild brain injury [22]. We also showed that CCL5 contributes to hippocampal neurons’ ATP generation and synaptic complex formation [1]. In addition, CCL5 expression after spinal cord injury is associated with axon regeneration and immune suppression [57]. The plasma level of CCL5 is highly correlated with BDNF, EGF (epidermal growth factor), and VEGF (vascular endothelial growth factor) after stroke [51]; those trophic factors contribute to neuron growth and proper brain function. Taken together, these findings suggest that CCL5 can help neurons survive from energy shortage and promote axon and synapse regrowth after injury.

In the present study, we identified that this chemokine has a robust and attractive effect on neuron growth cone formation, axon growth and myelination during post-injury repair. We specifically demonstrate for the first time that these effects are related to the ability of neuronal CCL5 to promote axon growth cone formation, axonogenesis and myelination through P70S6/mTOR signaling and the NRG1/ErbB and FGF pathways, ultimately augmenting neuronal axon and synapse regrowth after injury. Together, CCL5 promotes recovery of axogenesis and neurogenesis after brain injury.

## Materials and methods

### Animas and weight-drop (WD) model of mild TBI and behavioral tests

The Institutional Animal Care approved traumatic brain injury (TBI) study protocols and Use Committees of the Taipei Medical University (Protocol numbers: LAC-2021-0004; LAC-2021-0360). Male C57BL/6J mice were provided by the National Laboratory Animal Center (NLAC), NARLabs, Taiwan. B6.129P2-Ccl5^tm1Hso^/J mice (CCL5 knockout, CCL5-KO, Stock No: 005090), purchased from Jackson Laboratory, were maintained in NLAC. 3–5 mice were housed per cage with free access to food and water. Animal rooms were kept on a 12:12-hour light/dark cycle with a room temperature of 25 °C; behavior tests were also performed at 25 °C Only male mice were used in this study because of the well-documented neuroprotective actions of estrogen in several neurodegeneration models in mice [11, 12], which would add a confound here. Estrogen can inhibit the activation of microglia and astrocyte related neuroinflammation after injury [55]. On the other hand, estrogens also promote neurogenesis and neural recovery [47]. These functions of estrogen would interfere with the current study about chemokine CCL5. CCL5 is a chemokine which has strong effects on microglia and astrocyte; our previous study also showed that CCL5 can promote neurite outgrowth and synaptic complex formation [1, 8]. Thus, we avoided the effects of estrogen in the current study.

**Fig. 1:**
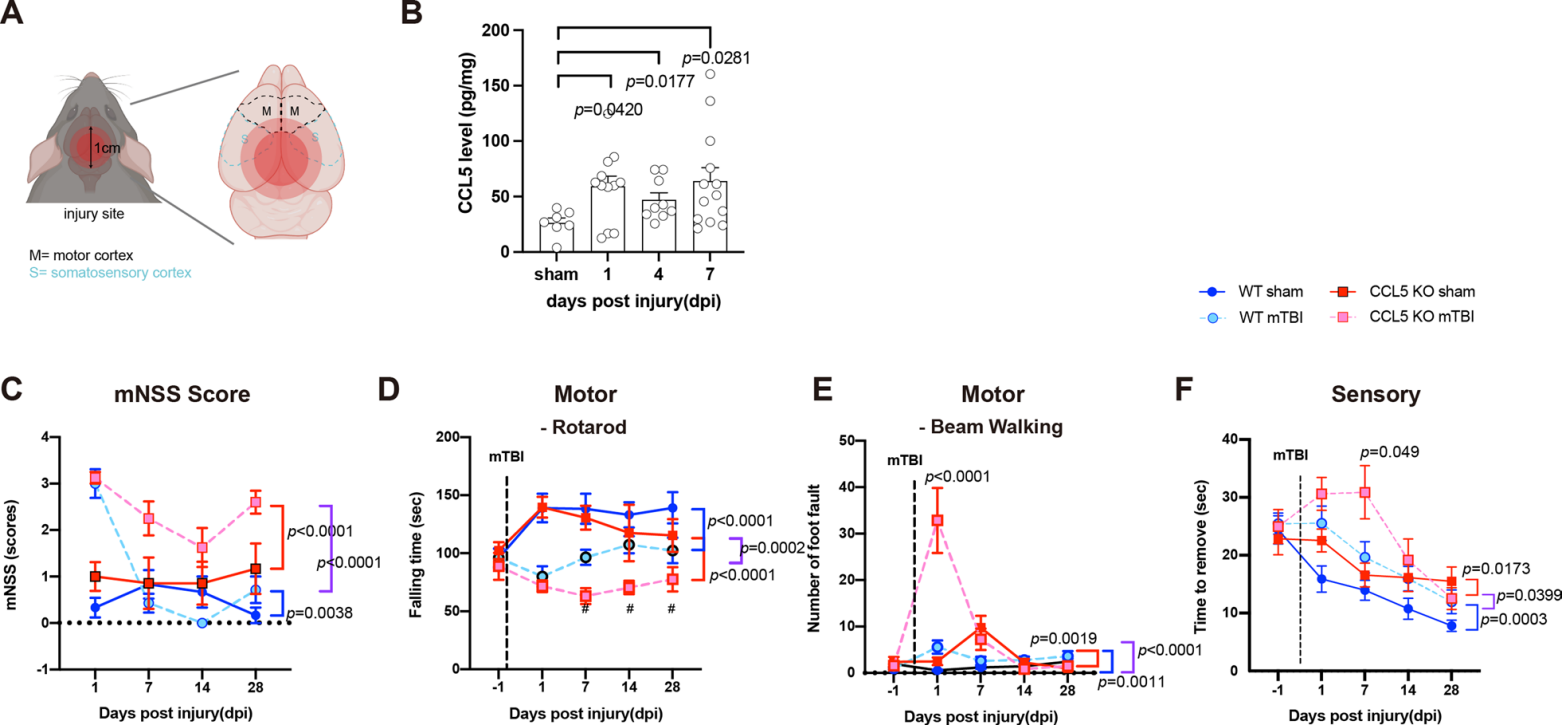
Weight drop induced mild traumatic brain injury and caused cortical function impairment in mice. **A** An illustration of the weight-drop-induced motor and sensory cortex injury site. **B** The protein levels of CCL5 in the cortex after 1-, 4-, and 7 days of injury (dpi) were detected by ELISA assay (sham vs. 1 dpi, *p* = 0.0420; sham vs. 4 dpi, *p* = 0.0177; sham vs. 7dpi, *p* = 0.0281. Data were presented as mean ± SEM and analyzed by *t*-test following Mann-Whitney test). **C** The mNSS score of both WT and CCL5-KO mice showed mild brain injury. (WT sham vs. TBI, *p* = 0.0038; KO sham vs. TBI, *p* < 0.0001; WT-TBI vs. KO-TBI, *p* < 0.0001). The motor function of 4 groups of mice included falling time from the accelerating Rotarod (WT sham vs. TBI, *p* < 0.0001; KO sham vs. TBI, *p* < 0.0001; WT-TBI vs. KO-TBI, *p* = 0.0002) (**D**) and foot faults with beam walking (WT sham vs. TBI, *p* = 0.0011; KO sham vs. TBI, *p* = 0.0019; WT-TBI vs. KO-TBI, *p* < 0.0001) (**E**). Sensory function was analyzed by limb sticker removal (WT sham vs. TBI, *p* = 0.0003; KO sham vs. TBI, *p* = 0.0173; WT-TBI vs. KO-TBI, *p* = 0.0399) (**F**). Data was analyzed from both paws. (*n* = 7–9 animals in **C–F**) Data in **C–F** were analyzed by two-way ANOVA and presented as mean ± SEM. The time of induced brain injury (mild traumatic brain injury, mTBI)

TBI was induced by weight drop as previously described [5, 22, 40–42]. Mice were first anesthetized with 2.5% isoflurane with an airflow (rate:1.5–2.0 l/mins), then placed on a foam sponge underneath the weight-drop device (a hollow cylindrical tube with an inner diameter of 1.2 cm, 100 cm height). Mild TBI was induced by a 30 g weight to strike at the center of the mouse brain (Fig. 1A). Sham animals were only anesthetized by isoflurane without TBI induction. Body temperature was maintained during surgery and recovery at 38ºc using a heated pad and incubator.

CCL5 KO mice were treated with recombinant mouse CCL5/RANTES Protein (478-MR-025, R&D system) in the rescue experiments. CCL5 recombinant protein was diluted with PBS and given to CCL5 KO mice at a 300 µg/kg dosage through intranasal delivery as described previously [22]. In the post-treatment group, CCL5 administration persisted in CCL5 KO mice at a dosage of 30 µg/kg, commencing after 3 days post-infection (dpi) and continued every two days for one month. CCL5 recombinant protein was conjugated by Alexa Fluor™ 594 (Microscale Protein Labeling Kit, A30008, Invitrogen) for tracking the distribution of recombinant CCL5 in the brain [22].

- *mNSS Score*: Modified Neurological Severity Scores (mNSS) is a neurological function test with several behavioral parameters that indicate overall neuronal function. mNSS scores include motor, sensory, reflex and balance performance evaluation. The parameters were graded as 0 to 18; a composite score of ≤ 6 designates normal or a mild TBI, 7–12 designates a moderate TBI, and ≥ 13 designates a severe TBI.
- *Beam walking*: The beam consists of 1.2-meter beam with a flat surface of 10 mm (width) x 15 cm (height). A black “safe box” was placed at the end of the beam. Mice went through the beam three times; the first time was taken as training; the last two times were analyzed with the number of foot faults. The time spent passing the beam was also counted [39].
- *Rotarod*: An accelerating rotarod (Ugo-Basile, Collegeville) [54] was used. Mice were placed on the slowly rotating rod (5 rotations per minute, RPM) for training, then switched to an acceleration mode from 5 to 60 rpm within 3 min. Mice performed three trials and the time of falling from the rod in those trials was recorded.
- *Adhesive removal test (ART)*: Small adhesive tape strips (0.3 cm x 0.4 cm) were placed on both the left and right paws of the mice; they were then put into an empty home cage, and the time for removing tape strips was recorded. Observers were blinded to treatment groups in all behavioral tests [6].

### Golgi staining and immunohistochemistry staining

Mice were anesthetized with Zoletil-50 (66F4, Virbac) and Rompum (PP1523, Bayer) mixture and perfused with 0.9% NaCl for Golgi staining or perfused with 4% paraformaldehyde (Sigma, 158127) in 0.1 M phosphate buffer (0.1 M PB) for immunostaining. The mouse whole brain was treated with the impregnation solution provided in the FD Rapid GolgiStain™ Kit (FD Neurotechnologies, Inc., Columbia, USA, Cat. #: PK401), as we previously published [1]. In short, brains were sectioned into 150 μm slices with a Microtome (Leica, VT1000S) and then developed using the FD Rapid GolgiStain solution following the experimental procedure provided by the manufacturers. Stained brain slides were then fixed on glass slides coated with gelatin solution (Merck cat no:1.04070) dehydrated with EtOH and Xylene and mounted by Acrytol mounting medium (3801720, Leica). For the Sholl analysis, cortical neurons (40x magnification) were captured and processed using Fiji-ImageJ2 (NIH, v2.9). Individual neurons were assessed using concentric circles with increasing radii (1 μm increments) centered on the cell body. Quantification of spines and swelling dendrites was analyzed using Fiji-ImageJ2. The number of spines was normalized by the length of the axon, and the percentage of swelling dendrites was calculated. Quantification was conducted utilizing data derived from 5 slides from 3 individual mice within each experimental group.

For immunostaining, mouse brains were cryostat sectioned at 30 μm; brain sections were treated with 1% sodium bicarbonate in 0.1 M PB for 30 min and blocked with 3% normal goat serum (NGS) and 3% BSA in PBS containing 0.25% Triton X-100. Primary antibody was prepared in a blocking buffer and incubated with brain sections at 4℃ overnight. After washing three times with PBS with calcium and magnesium (PBSc/m), brain tissues were incubated with secondary Ab for 40 min at room temperature. DAPI (Sigma, D9542) was used to label the nuclei. Tissues were mounted on slides with antifade mounting medium VECTASHIELD (Vector H1000). Primary antibodies were listed in the antibodies table-Supplementary Table 1. Controls consisted of omission of the primary antibody, and observers were blinded as to the treatment group. Quantification was performed using data obtained from analysis of 3–4 slides derived from 3 individual mice within each experimental group.

### Western blot protein analysis

Mouse cortex tissues were isolated and lysed by RIPA buffer (Millipore, # 20-188, Temecula, CA, USA.) with protease-phosphatase inhibitor (Thermo Scientific, #78446, IL, USA). The protein concentration was determined by Bradford BCA assay (Bio-Rad, 500-0006). Electrophoresis was performed with a 10% Tris-HCl protein gel; 30 µg of total protein was loaded per lane and transferred to PVDF membranes by a Trans-Blot^®^ Cell system (Bio-Rad). Membranes were blocked by 5% skim milk in Tris-buffered saline (TBS) with 0.05% Tween 20 (TBST) for 1 h at room temperature. Membranes were incubated with different primary antibodies (listed in the table) at below 4 °C overnight, then washed 3 times with TBST and incubated with HRP-conjugated secondary antibodies for 1 h at room temperature. After extensive washing with TBST, membranes were developed by the Clarity Western ECL Substrate kit (Bio-Rad, 1705061); the intensities of protein bands were quantified by Fiji-imageJ2 (NIH, v2.15). Quantification was conducted based on the analysis of 4 to 6 mice within each respective experimental group. Antibodies used are listed in the Supplementary Table 1.

### CCL5 ELISA

The levels of CCL5 in mouse brain tissues were measured using the mouse CCL5/RANTES DuoSet ELISA kit (DY478, R&D System) following the manufacturer’s protocol as in our previous studies [8, 22]. In short, 96-well microplates (Corning, 9018) were coated overnight with capture antibody at room temperature (RT). After 3 washings with PBS containing 0.05% Tween 20 (PBST), plates were incubated with blocking buffer (1% BSA in PBS) for 2 h at room temperature (RT) and washed with PBST. 100 µl of brain tissues were added to microplates, incubated for 2 h at RT, and washed with buffer 3 times. A detection antibody was added and set for 2 h, then streptavidin-HRP plus substrate for signal development for 30 min. Microplate samples were measured with an absorbance reader at 450 nm by iMARK Microplate reader (BioRad). The level of CCL5 in each sample was calculated based on a standard curve prepared for the same experiment and normalized by protein concentration. Quantification was conducted based on the analysis of 4 to 6 mice within each respective experimental group.

#### LC-MS/MS and proteomic analysis

- ***Sample preparation***: Cortical tissues were isolated from CCL5^−/−^ sham (*n* = 3), CCL5^−/−^ TBI (*n* = 3), CCL5^−/−^ TBI + CCL5 pretreatment (*n* = 5), and CCL5^−/−^ TBI + CCL5 posttreatment (*n* = 4) groups of mice at 28dpi. The tissue was homogenized and extracted for protein with ice-cold PBS. Pre-cold acetone was mixed with the protein solution (acetone to protein solution ratio was 4:1) and stored at − 80 °C for at least 2 h; samples were then centrifuged at 15,000 *g* for 15 min at 4 °C. The protein pellet was washed twice with 80% acetone solution (4:1 diluent with ddH_2_O) and precipitated by 15,000 *g* centrifugation for 10 min at 4 °C during each wash. The supernatant was discarded, and the pellet was air-dried on ice and resuspended with 6 M urea solution. Protein concentration was measured with Barford assay reagent (5000006, Bio-Rad, USA); 50 µg of total protein from each sample used in the LC-MS/MS analysis was studied at the proteomics core in National Taiwan University (NTU) - mass spectrometry technical research services from Consortia of Key Technologies and Instrumentation Center (Taipei, Taiwan). The identified proteins were next analyzed by DAVID Bioinformatics Resources 6.8. for gene ontology (GO) and Ingenuity Pathway Analysis (IPA) for pathway, network, and upstream molecule identification.
- ***The Database for Annotation***,*** Visualization***,*** and Integrated Discovery (DAVID) analysis***: Samples were divided into four sets of comparisons, including (1) CCL5^−/−^ sham vs. CCL5^−/−^ TBI, (2) CCL5^−/−^ TBI vs. CCL5^−/−^ TBI + CCL5 pretreatment, (3) CCL5^−/−^ TBI vs. CCL5^−/−^ TBI + CCL5 post-treatment, and (4) CCL5^−/−^ TBI + CCL5 pretreatment vs. CCL5^−/−^ TBI + CCL5 post-treatment. The fold change of identified proteins > 1.25 or < 0.75 and unpaired *t*-test *p*-value < 0.05 from each comparison set were chosen for further analysis. In DAVID analysis, proteins were analyzed according to biological process, cellular component, molecular function (3 categories), and KEGG pathway; *p*-value < 0.05 are shown for each study.
- ***Ingenuity Pathway Analysis (IPA)***: The selection protein ID and log2 fold-change (FC) were uploaded to IPA (Qiagen) and analyzed with core analysis (default setting). Core analysis includes canonical pathways, upstream analysis, disease and function, and protein network prediction.

###  Primary cortical neuron culture and recombinant CCL5 treatment

Primary neurons were cultured from C57BL/6 and CCL5^−/−^ embryos at day 16.5–17 (E16.5-17) [1, 22]. Embryonic brain tissues were digested with buffer (2 mg/ml papain, Worthington, LS003119) and 0.05% Trypsin-EDTA (Gibco, 25200-072) in Dulbecco’s Modified Eagle Medium (DMEM, 12800-017, Gibco) for 14 min and seeded with plating medium (neurobasal medium, 21103-049, Gibco) containing 10% v/v heat-inactivated fetal bovine serum (FBS, 10437-028, Gibco), 1% v/v Antibiotic-Antimycotic (15240-062, Gibco), and 2mM L-glutamine (25030, Gibco)). After a 2 h attachment, the medium was replaced with complete medium (neurobasal medium containing 1% v/v N-2 supplement, 17502048, Gibco), 2% v/v B-27 supplement (17504044, Gibco), 1% v/v Antibiotic-Antimycotic and 2mM L-glutamine). Half of the medium was replaced every 3 days. For the growth cone study, 1 × 10^5^ primary cortical neurons were seeded in a 6-well plate with a poly-L-lysine coated coverslip. Cells were treated with CCL5, CCR5 inhibitor (Maraviroc, MCE, Cat. No.: HY-13004), mTOR inhibitor (Rapamycin, PHG0026, ThermoFisher), or ROCK inhibitor (Y27632, Taiclone) at DIV6 and fixed with 4% PFA + 3.7% sucrose in PBS at DIV7.

### Immunocytochemistry staining and image processing

Primary cortical neurons were fixed with % PFA + 3.7% sucrose in PBS, then incubated with 50 mM NH_4_Cl for 10 min and blocked with a blocking buffer (3% BSA, 10% TX-100 in PBS) for 60 min. After extensive wash with PBS 3 times, neurons were incubated with the primary antibody diluted in blocking buffer overnight at 4 °C and the corresponding secondary antibody and Phalloidin-594 (A12381, Invitrogen) for 1 h at room temperature the next day. Finally, coverslips were mounted with Fluoromount™ Aqueous Mounting Medium (F4680, Sigma-Aldrich). Images were acquired with the STELLARIS 8 confocal microscope (Leica). The exported images were imported into ImageJ-Fiji (V2.9.0), and the areas of Phalloidin + and Tuj-1 + were analyzed. The Phalloidin + area was normalized by the Tuj-1 + area, and the result was compared with the WT untreated group to determine the reactive Phalloidin intensity.

### Quantitative PCR

Injured cortical tissue including motor and sensory cortex from both wild-type (WT) and CCL5^−/−^ mice were harvested and extracted the total RNA by TRIZOL reagent (15596018, Invitrogen) Tissue RNA subsequent reverse transcript into cDNA using the High-Capacity cDNA Reverse Transcription Kit (4368813, Applied Biosystems™). Quantitative PCR analysis was carried out by iTaq Universal SYBR Green Supermix (Bio-Rad) with the StepOnePlus™ Real-Time PCR System (4376600, Applied Biosystems™, USA). Primer sequence for mouse CXCR4 were as: forward − 5’-TGGAACCGATCAGTGTGAGT-3’; reverse − 5’-AACCCATGACCAGGATCACC-3’. GADPH primers were as: forward − 5’-GTGTTCCTACCCCCAATGTGT-3’; reverse − 5’-AGAGTGGCTGTGTGTTGAAG-3’.

### Statistical analysis

Statistical analysis was conducted using GraphPad Prizm 8.0 (GraphPad Software, Dan Diego, CA, USA). An unpaired t-test analyzed the differences between the two groups; One-way ANOVA analyzed the same group analysis with a confidence interval of 95%, and multiple factors analysis was conducted with two-way ANOVA. The Bonferroni correction was used for serial measurements. A *p*-value < 0.05 was considered significant. All results are presented as mean *±* SEM.

## Results

### Weight-drop model of mild brain injury caused motor and sensory cortex dysfunction in CCL5 knockout (CCL5-KO) mice

Both wild-type (WT, C57BL/6) and CCL5-KO mice were given mild brain injury using a weight-drop (WD) system at about two months of age as in our previous study [22]; the weight drop induced head injury as shown in Fig. 1A. The redlined circled impact area includes the mouse motor and sensory cortex. The expression level of CCL5 in WT mouse cortical tissues was about 40 pg/mg in the cortex, which rapidly increased to 60–160 pg/mg after 1-, 4-, and 7-day post-injury (dpi) by ELISA assay (Fig. 1B). The mNSS was used to evaluate neurological injury after 1-, 7-, 14-, and 28 days after injury. The mNSS score was lower than 4 in all four groups at all time-points consistent with a mild injury in mice. The mNSS score slightly increased at 1 dpi and was reduced to 1 at 7 dpi in the WT-mTBI group, indicating a functional recovery in WT mice. In contrast, the mNSS score was not reduced in CCL5-KO mice even one month after brain injury (Fig. 1C). Motor cortex function and sensory function were evaluated by Rotarod, beam walking, and limb adhesive removal tests (ART). The coordination and balance of mice on the Rotarod (time falling from Rotarod) after mTBI was significantly reduced in both WT and KO mice at 1 dpi (Fig. 1D). As with mNSS, the balance performance on Rotarod improved in the WT mTBI group of mice but not in CCL-KO mice after 7 dpi (Fig. 1D). The number of foot faults on beam walking was slightly increased in WT mice but markedly increased in CCL5-KO at 1-dpi (Fig. 1E). This parameter improved after 7–14 dpi in both groups of mice (Fig. 1E). The walking time over the beam was 10.25 ± 0.716 s in WT sham and 14.67 ± 0.99 s in WT mTBI groups at 1 dpi (*p* = 0.002); in KO mice, the walking time at 1-dpi was 12.65 ± 1.36 s in the sham group and 24.62 ± 2.73 s in the TBI group (*p* = 0.002, compared to sham; *p* = 0.0082 compared to WT TBI group). The time to remove adhesive stickers on the paws also increased in both WT and KO mice after mTBI, which improved more slowly in CCL5-KO mice after 7–14 dpi compared to 1–7 dpi in WT mice (Fig. 1F). Taken together, the delayed recovery suggests expression of CCL5 is important for neural functional recovery after mild TBI.

### The recovery of axonal injury and synapse reformation was impaired in the CCL5-KO cortex after mild TBI

**Fig. 2:**
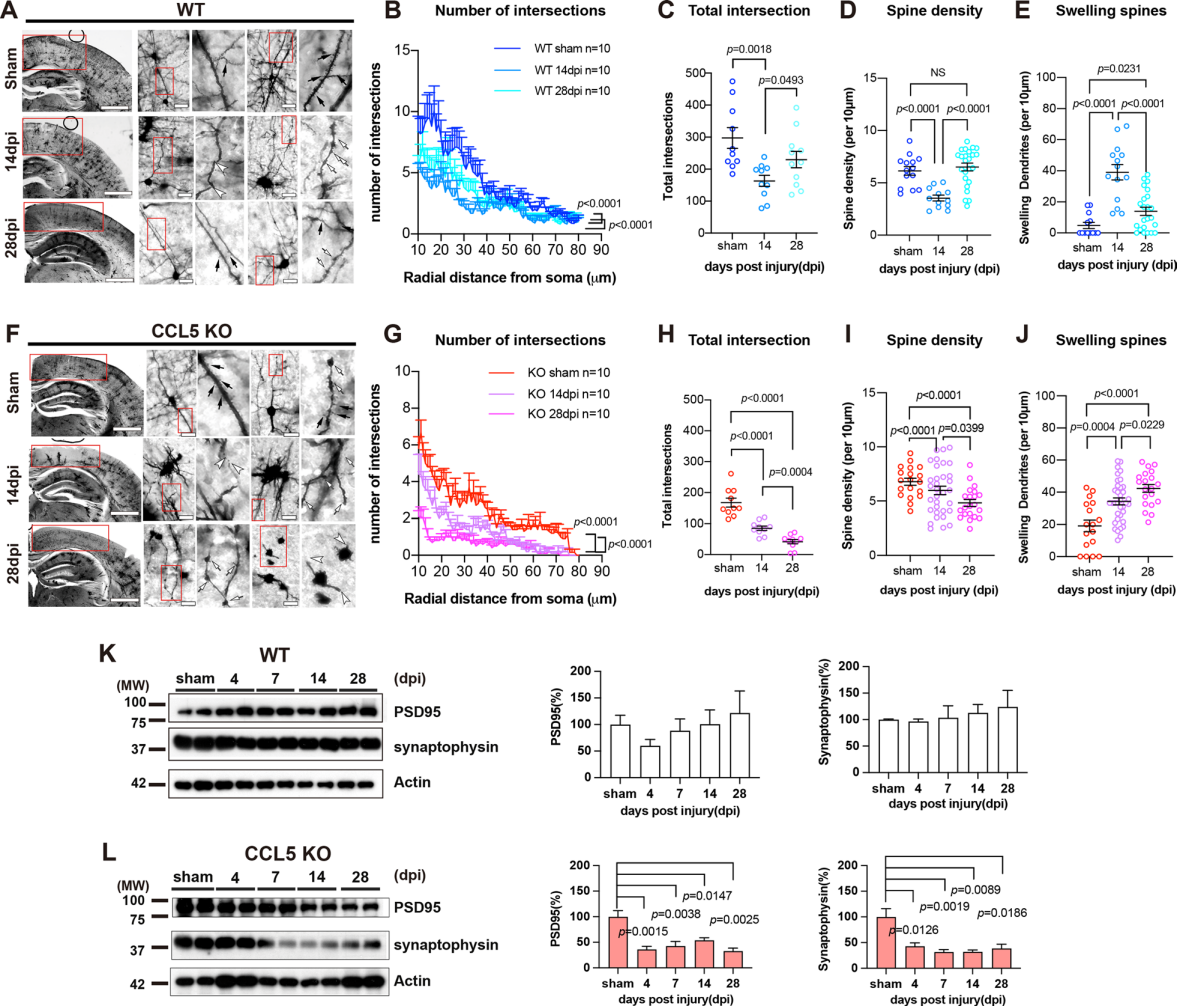
The recovery of axonal injury was impaired in the CCL5-KO cortex after mild TBI. Golgi staining revealed the axon and spine structures in WT and CCL5-KO mouse cortex with sham treatment and mild TBI – 14 and − 28 days of injury (dpi). **A**,** F** The representative images of neurites and spine structures in WT and CCL5-KO mouse cortex; boxed regions were enlarged on the right. Black arrowheads point to the normal dendritic spines, and white arrows point to swollen neurites and spines. Scale bar = 1 mm and 20 μm. **B**,** G** The number of intersections (WT sham vs. 14 dpi, *p* < 0.0001; WT 14 dpi vs. 28 dpi, *p* < 0.0001; KO sham vs. 14 dpi, *p* < 0.0001; KO 14 dpi vs. 28 dpi, *p* < 0.0001. Data were analyzed by two-way ANOVA and presented as mean ± SEM), **C**,** H** total intersections (WT sham vs. 14 dpi, *p* = 0.0018; WT 14 dpi vs. 28 dpi, *p* = 0.0493; KO sham vs. 14 dpi, *p* < 0.0001; KO 14 dpi vs. 28 dpi, *p* = 0.0004.), **D**,** I** spine density (WT sham vs. 14 dpi, *p* < 0.0001; WT 14 dpi vs. 28 dpi, *p* < 0.0001; WT sham vs. 28 dpi, no significant difference, NS; KO sham vs. 14 dpi, *p* < 0.0001; KO 14dpi vs. 28 dpi, *p* = 0.0399; KO sham vs. 28 dpi, *p* < 0.0001.), and **E**,** J** swollen spines (WT sham vs. 14 dpi, *p* < 0.0001; WT 14 dpi vs. 28 dpi, *p* < 0.0001; WT sham vs. 28 dpi, *p* = 0.0231; KO sham vs. 14 dpi, *p* = 0.0004; KO 14dpi vs. 28dpi, *p* = 0.0229; KO sham vs. 28 dpi, *p* < 0.0001.) were quantified in different groups of mice (*n* = 10 in each group). Data in **C–E** and **H–J** were analyzed by unpaired *t*-test and presented as mean ± SEM. **K**,** L** The expression of synaptic proteins – PSD95 and synaptophysin in different groups of WT and CCL5-KO mouse cortex, including sham, 4, 7, 14, and 28 dpi, was analyzed by western blot. Quantification of results from 3 independent mouse samples in each group is listed above the images of protein blots in 2 **K–L**. (KO PSD95: sham vs. 4 dpi, *p* = 0.0015; sham vs. 7 dpi, *p* = 0.0038; sham vs. 14 dpi, *p* = 0.0147; sham vs. 28 dpi, *p* = 0.0025. KO Synaptophysin: sham vs. 4 dpi, *p* = 0.0126; sham vs. 7 dpi, *p* = 0.0019; sham vs. 14 dpi, *p* = 0.0089; sham vs. 28 dpi, *p* = 0.0186. Data were presented as mean ± SEM and analyzed by *t*-test following Mann-Whitney test)

Golgi staining was used to show the neurite structures in cortical neurons adjacent to the injured region (Fig. 2A, F). The number of intersections from soma and total intersections of neurites were evaluated by Sholl analysis. The number of intersections, total intersections, and spine density were reduced, and the number of swollen spines was increased in WT mice after 14 days of injury (Fig. 2B–E) when cortical function performance had already recovered. Those parameters were improved after 28 days as enhanced numbers of intersections, total intersections, and spine density (Fig. 2B–D); the swollen spines were also reduced after 28 days (Fig. 2E). In contrast, the number of intersections, total intersections and the spine density continued to be reduced in CCL5-KO mouse cortex after mTBI over 14 to 28 dpi (Fig. 2G–I), and the number of swollen spines also continually increased (Fig. 2J). The level of synaptic proteins—PSD95 reduced slightly and synaptophysin levels reduced and were maintained in CCL5-KO mouse cortex tissue over 14 to 28 dpi (Fig. 2L); this was not seen in the WT cortex after injury (Fig. 2K).

Taken together, these findings suggest that impairment of cortical motor and sensory function in CCL5-KO mice might result from the loss of neurites and spines after mTBI. The repair of neurites and regrowth of spines was manifested around post-injury 14–28 days according to Golgi staining in WT, and this repair process may be compromised by lack of CCL5 in the KO animals.

#### Reduced activation of axonogenesis, synaptogenesis, and myelination signaling molecules in CCL5-KO cortex after mTBI

**Fig. 3:**
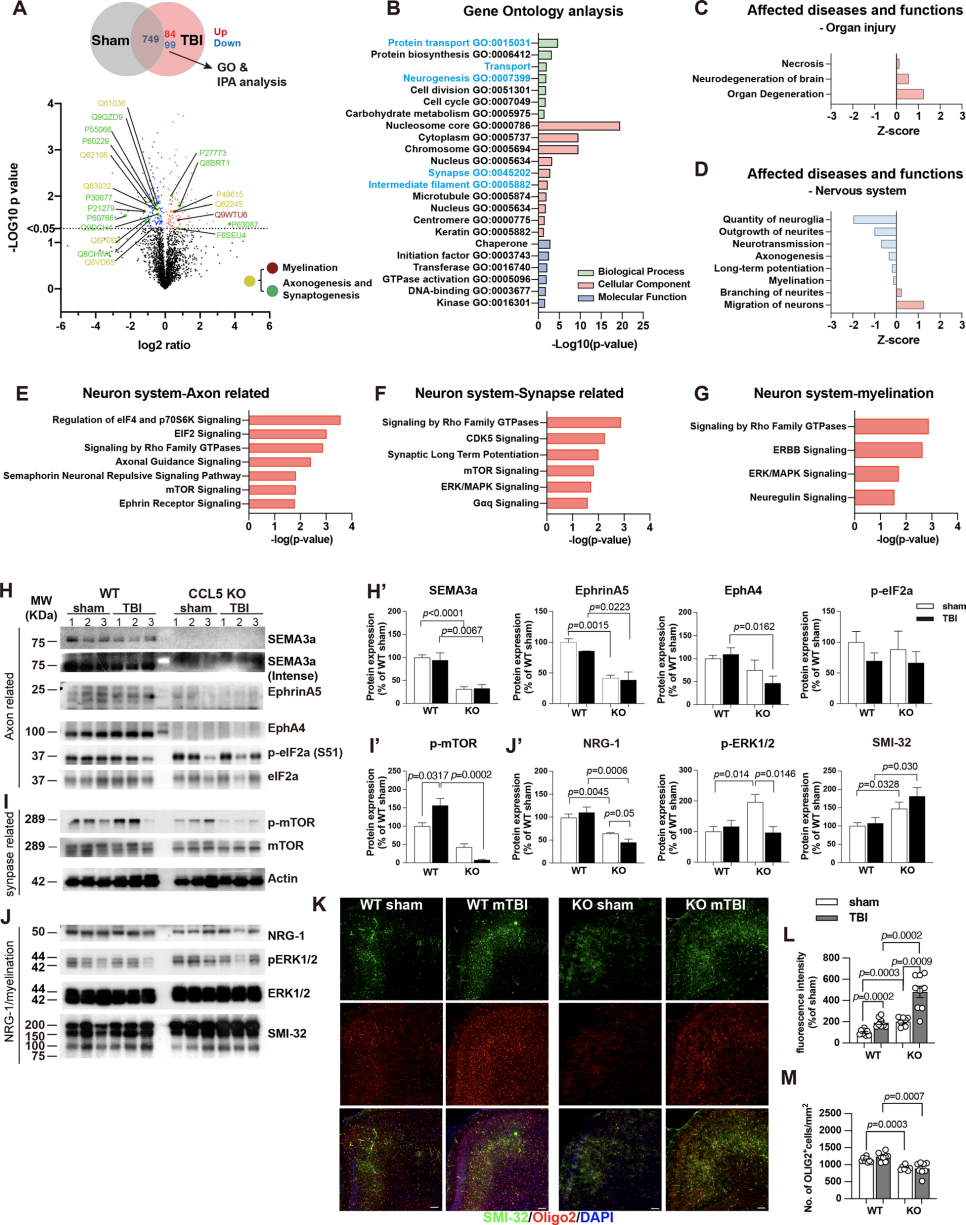
LC-MS/MS analysis identified a reduction of axonogenesis and myelination signaling pathways in CCL5-KO mice with mild brain injury. **A** Venn diagram comparing DEPs (differentially expressed proteins) and volcano plot of significant DEPs between sham and mild TBI CCL5-KO mouse cortex. DEPs: p-value < 0.05 in comparison to sham control, respectively. Colored points represent log2 ratio > 0 upregulated protein (red) and log2 ratio < 0 downregulated protein (blue). Selected axonogenesis and myelination pathway-related proteins are highlighted as indicated (Red: myelinations, Green: axonogenesis and synaptogenesis, Yellow: overlapping). Results from Gene Ontology (GO) enrichment analysis of 183 identified proteins (84 up regulated, 99 down regulated) against a background list of all known mouse protein symbols. **B** Identified GO terms from the three GO groups are shown (Green bar: biological process, Red bar: cellular component, Blue bar: molecular function. Blue character: neuron function related). The strength of enrichment of each GO term was indicated by the Log10 p-value (X-axis). **C**, **D** IPA analysis identified affected diseases and function in organ injury (**C**) and nervous system (**D**) categories. Z-score values indicated that functions are predicted to be activated (red) or inhibited (blue). Selected IPA canonical pathways in the nervous system identified significant DEPs related to axon (**E**), synapse (**F**), and myelination signaling pathways (**G**). Protein blots analyzed the expression of identified proteins in sham and mTBI groups of WT and CCL5-KO mouse cortical tissue, including **H, H'** axon guidance-related Sema3a (WT sham vs. KO sham, *p* < 0.0001; WT TBI vs. KO TBI, *p* = 0.0067), EphrinA5 (WT sham vs. KO sham, *p* = 0.0015; WT TBI vs. KO TBI, *p* = 0.0223), EphA4 (WT TBI vs. KO TBI, *p* = 0.0162), and p-EIF2/EIF2; **I, I'** synapse-related p-mTOR/mTOR (WT sham vs. TBI, *p* = 0.0319; WT TBI vs. KO TBI, *p* = 0.0002), and **J, J'** myelination-related: NRG-1 (WT sham vs. KO sham, *p* = 0.0045; KO sham vs. KO TBI, *p* = 0.05; WT TBI vs. KO TBI, *p* = 0.0006), p-Erk/Erk (WT sham vs. KO sham, *p* = 0.014; KO sham vs. KO TBI, *p* = 0.0146), and SMI32 (WT sham vs. KO sham, *p* = 0.0328; WT TBI vs. KO TBI, *p* = 0.030). **K,** The immunostaining of unmyelinated neuritis with SMI32 antibody (green) and oligodendrocyte by Oligo2 antibody (red) in different groups of mouse cortex. DAPI, blue, for nucleus. Scale = 100 μm. The quantification of **L** SMI-32 and **M** Oligo-2 in sham and mTBI groups of WT and KO mice (SMI32: WT sham vs. KO sham, *p* = 0.0003; WT sham vs. TBI, *p* = 0.0002; KO sham vs. TBI, *p* < 0.0009; WT TBI vs. KO TBI, *p* = 0.0002) (Oligo2: WT sham vs. KO sham, *p* = 0.0003; WT TBI vs. KO TBI, *p* = 0.0007). Data were analyzed by unpaired *t*-test

To identify the protein profile changes in the damaged brain and its correlation with axon injury in CCL5-KO mice after mild TBI, tissues from the CCL5-KO sham and injured mouse cortex were harvested and then analyzed by LC-MS/MS. Gene Ontology (GO) enrichment analysis of the entire list of 932 proteins was carried out and identified 183 proteins, including 84 up-regulated and 99 down-regulated proteins (adjusted p-value < 0.05) (Fig. 3A and Supplementary Fig. 1A, also see Supplementary Data file 1); identified GO terms for each category are shown in Fig. 3B and Supplementary Data File 2. Synapse and intermediate filament-related proteins were identified in the cellular component category (Fig. 3B, blue characters). Ingenuity Pathway Analysis (IPA) revealed that the major affected disease and function moieties were associated with cell morphology, organization, and development; organ degeneration, neurodegeneration, and necrosis were increased in the Organ injury category (Fig. 3C, Supplementary Fig. 1B). Nervous system development and function were also the major affected disease and function categories; an outgrowth of neurites, axonogenesis, and myelination were reduced in the Nerve system category (Fig. 3D, Supplementary Fig. 1B). Canonical pathways analysis of nervous system subcategories identified signaling pathways primarily associated with axonogenesis, axon guidance, and neuritogenesis (Supplementary Fig. 1C). These were EIF2, eIf4, and p70S6K, Rho family GTPase, axonal guidance, CDK5, semaphorin, mTOR, Ephrin, and ERK/MAPK signaling (Fig. 3E, F). Neuregulin and ERBB signaling are myelination-related signaling molecules (Fig. 3G). Proteins identified in each pathway are listed in the Supplementary Data file 3. We detected the activation of axon guidance-related signaling molecules—Semaphorin 3A, EphinA5, EphA4, and eIF2a, which were significantly lower in the CCL5-KO groups of mice (Fig. 3H, H’). Downstream signaling molecules—phosphorylation of mTOR (S2448), which is also involved in synaptogenesis, was increased in the WT-TBI group but reduced in the CCL5-KO TBI group by western blot analysis (Fig. 3I, I’). This suggests that mTOR-related function in axonogenesis and synaptogenesis was impaired in CCL5-KO after injury.

Neuregulin, ErbB2/3, and Erk signaling are essential in promoting Schwann cell growth and survival, migration, extending axons, and myelination. The expression of Neuregulin and phosphorylated-Erk were reduced in the cortex tissue of the CCL5-KO TBI group of mice (Fig. 3J, J’); in contrast, SMI-32 labeled non-phosphorylated neurofilaments, which indicates damaged axons, was significantly increased in the injured cortex of CCL5-KO mice after 1 month (Fig. 3J–L, J’). Oligo2-labeled oligodendrocytes were raised in the injured cortex of WT mice after mTBI but not in the KO mice (Fig. 3K, M). Together, these data support our finding of axonal injury and synapse loss shown in Golgi staining (Fig. 2) and cortical neuron dysfunction (Fig. 1) after brain injury in KO mice.

CCL5 is an important chemokine in microglia activation, but the impact of inflammatory responses was not the major affected pathway (Supplementary Fig. 1B, green); the inflammatory response – macrophage activation and phagocytosis pathways were reduced in CCL5-KO mice after TBI (Supplementary Fig. 1E).

#### Intranasal delivery of recombinant CCL5 after injury improves both motor and sensory function

**Fig. 4:**
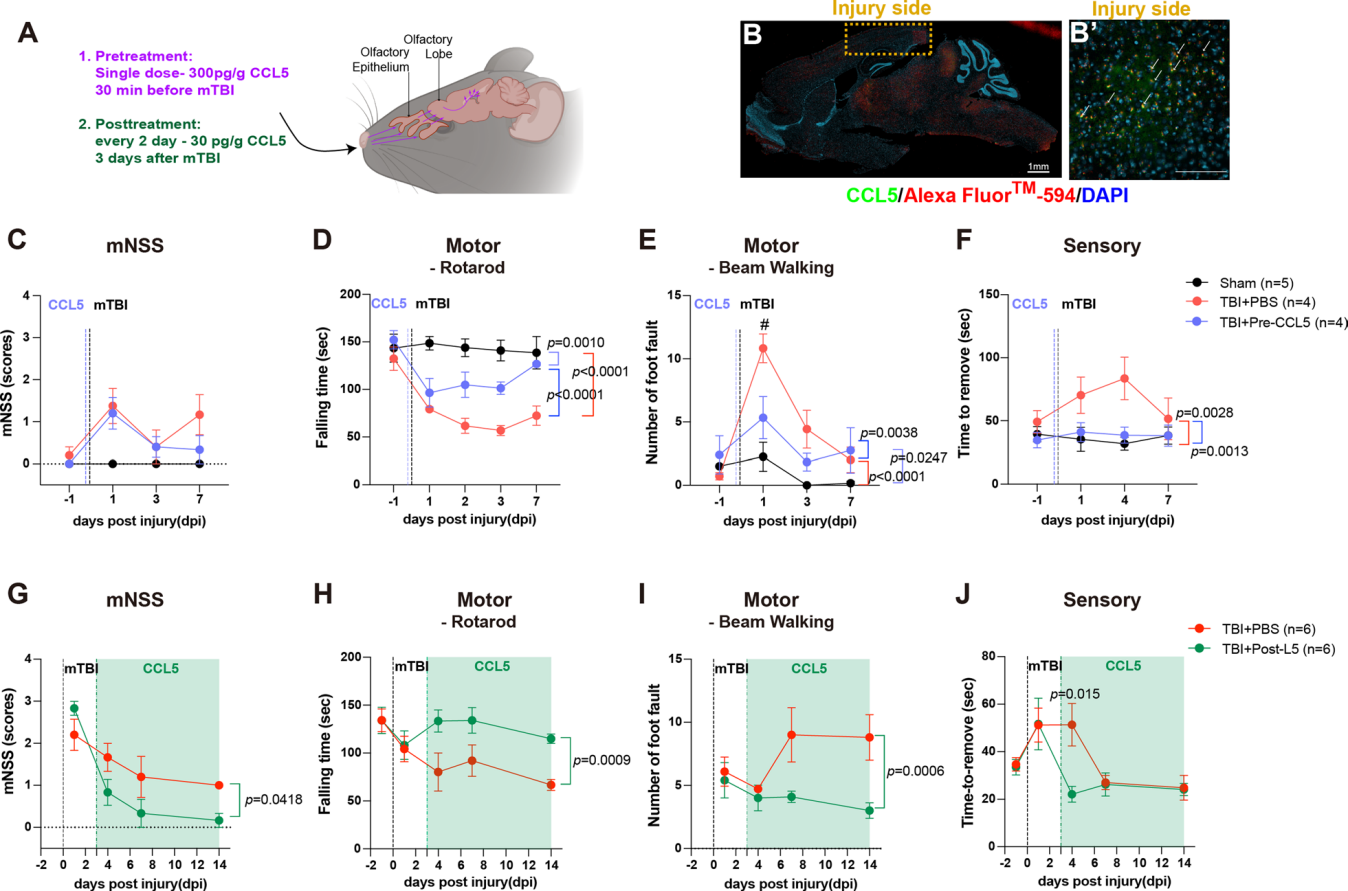
Cortical neuron dysfunctions were improved by intranasally delivering CCL5 into CCL5-KO mice. **A** An illustration of intranasal delivery (i.n.) of recombinant CCL5 into mice. Recombinant CCL5 was administered into mice either (1) 30 min before injury with a single dosage of 300 pg/g or (2) 3 days after injury with 30 pg/g every 2 days until 28 dpi. **B** Recombinant CCL5 conjugated with Alexa Fluor™ 594 was detected by Alexa Fluor™ 594 (red) and CCL5 specific antibody (green) in mouse cortex. Images of CCL5 at the injury site in the cortex were enlarged on the right **B**' (Scale bar = 1 mm in **B** and 100 µm in **B’**). DAPI labeled the nucleus. **C-J** A Black dashed line points to the time of brain injury. The Purple dashed line indicates the treatment with CCL5 before the weight drop impact in **C-F**; the green dashed line and green area indicate the post-treatment with CCL5 from 3 dpi until 28 dpi in **G-J**. **C**,** G** The mNSS score of the CCL5-KO sham group and mice treated with PBS (control) and CCL5 (300 pg/g, single dose) before mTBI or PBS (control) and CCL5 (30 pg/g, every two days) (**G**: PBS vs Post-L5, *p* = 0.0418) after mTBI. Motor function of CCL5-KO mice with i.n. PBS or CCL5 was analyzed by Rotarod **(D**,** H)** (**D**: sham vs PBS, *p* < 0.0001; sham vs Pre-L5, *p* = 0.0010; PBS vs Pre-L5, *p* < 0.0001. **H**: PBS vs Post-L5, *p*=0.0009), and beam walking (**E**,** I**), which was improved in both i.n. CCL5 treated groups. (**E**: sham vs PBS, *p* < 0.0001; sham vs Pre-L5, *p* = 0.0247; PBS vs Pre-L5, *p* = 0.0038. **I**: PBS vs Post-L5, *p* = 0.0006). **F**,** J** Sensory function was analyzed by sicker removal test (**F**: sham vs PBS, *p* = 0.0028; sham vs Pre-L5, NS; PBS vs Pre-L5, *p* = 0.0013. **J**: PBS vs Post-L5 at 4 dpi, *p* = 0.015, by *t*-test). The time to remove stickers in CCL5-KO mice was reduced after being treated with CCL5. (*n* = 4 ~ 5 in **C-F**; *n* = 6 in **G-J**). Data in **D-I** was analyzed by two-way ANOVA between groups and presented as mean ± SEM

As shown in Fig. 1B, the level of CCL5 protein increased to 60 ~ 160 pg/mg in cortex tissue after mild TBI; a low dose of CCL5 was thus used here in the following rescue experiments. To confirm the effect of CCL5 in neurite repair and synapse regrowth after brain injury, we studied intranasally (in) delivered recombinant CCL5 (rCCL5) into the mouse brain, as we previously used [22]. First, a single dosage of rCCL5 (300 pg/g) was administered into the CCL5-KO mouse brain just before inducing mTBI (Fig. 4A-1). However, a therapeutic treatment after an injury is also necessary for the clinical application; thus, we also administered a lower dosage series of rCCL5 (30 pg/g) into the CCL5-KO mouse brain 3 days after inducing mTBI every two days until 28 dpi (Fig. 4A-2). PBS treatment was used as a CCL5 TBI control. The rCCL5 was first conjugated with Alex Fluro^TM^-594 and the distribution of rCCL5- Alex Fluro^TM^-594 in the brain was detected by Alex Fluro^TM^-594 (red) as well as a CCL5-specific antibody in mouse cortex (Fig. 4B). The boxed region indicates the injured cortex region; B’ is part of an enlarged image from the boxed region (Fig. 4B). The tissue level of CCL5 was detected by CCL5 specific ELISA assay to confirm penetration efficiency. 40 pg/mg of CCL5 was detected in mouse prefrontal cortex (CTX) tissues after i.n. CCL5 for 6 h and was gradually reduced to 20 ~ 30 pg/mg of CCL5 after 24 h (Supplementary Fig. 2A), which is similar to the endogenous level of CCL5 in WT mouse cortex (Fig. 1B). The localization of rCCL5 in mouse cortex was co-labeled with the neuron marker-NeuN (Supplementary Fig. 2B), the astrocyte marker-GFAP (Supplementary Fig. 2C) and the microglia marker-Ibal (Supplementary Fig. 2D); the results showed that rCCL5 colocalized mostly with NeuN-positive neurons (~ 70%, Supplementary Fig. 2B, E).

In the pretreatment with CCL5 (PreL5) study, the neurological mNSS score was lower than 4 in all three groups of mice, including CCL5-KO sham, TBI + i.n. PBS, and TBI + i.n. CCL5 (PreL5) after 1-, 3- and 7-dpi (Fig. 4C). Motor function, assessed by Rotarod, improved in CCL5-treated CCL5-KO mice after 3 dpi (Fig. 4D); the foot faults also improved faster in mice receiving recombinant CCL5 compared to the PBS group (Fig. 4E). In ART - the time to remove paw stickers in CCL5-KO TBI mice receiving recombinant CCL5 was the same as in the CCL5-KO sham control animals (Fig. 4F). In contrast, the CCL5-KO TBI i.n. PBS group had a much slower time for sticker removal at dpi 1 and 4 (Fig. 4F). Thus, the cortical function parameters were improved within seven days in the PreL5 group of mice, which suggests early CCL5 intervention is beneficial for cortex neuron function recovery.

In the post-treatment of CCL5 (PostL5) study, the neurological mNSS score was also lower than 4 in TBI + i.n. PBS, and TBI + i.n. CCL5 (PostL5) at -3, -7, and − 14 dpi; the mNSS score was reduced faster in mice treated with CCL5 than in mice treated with PBS (Fig. 4G). The falling times from the Rotarod were reduced in both PBS and PostL5 groups of mice after mTBI, but which improved immediately when animals received rCCL5 (PostL5) but not PBS (Fig. 4H). The number of foot faults from the balance beam also improved after rCCL5 treatment but not PBS treatment (Fig. 4I). In ART - the time to remove paw stickers was also reduced quickly right after rCCL5 treatment (Fig. 4J).

These findings strongly support an important function of CCL5 in cortical neuronal recovery after mild brain injury. Both early intervention and subsequent treatment with CCL5 improved mouse motor and sensory function.

#### CCL5 treatment enhanced axonogenesis, synaptogenesis, and myelination signaling in mouse CCL5-KO cortex after mTBI

**Fig. 5:**
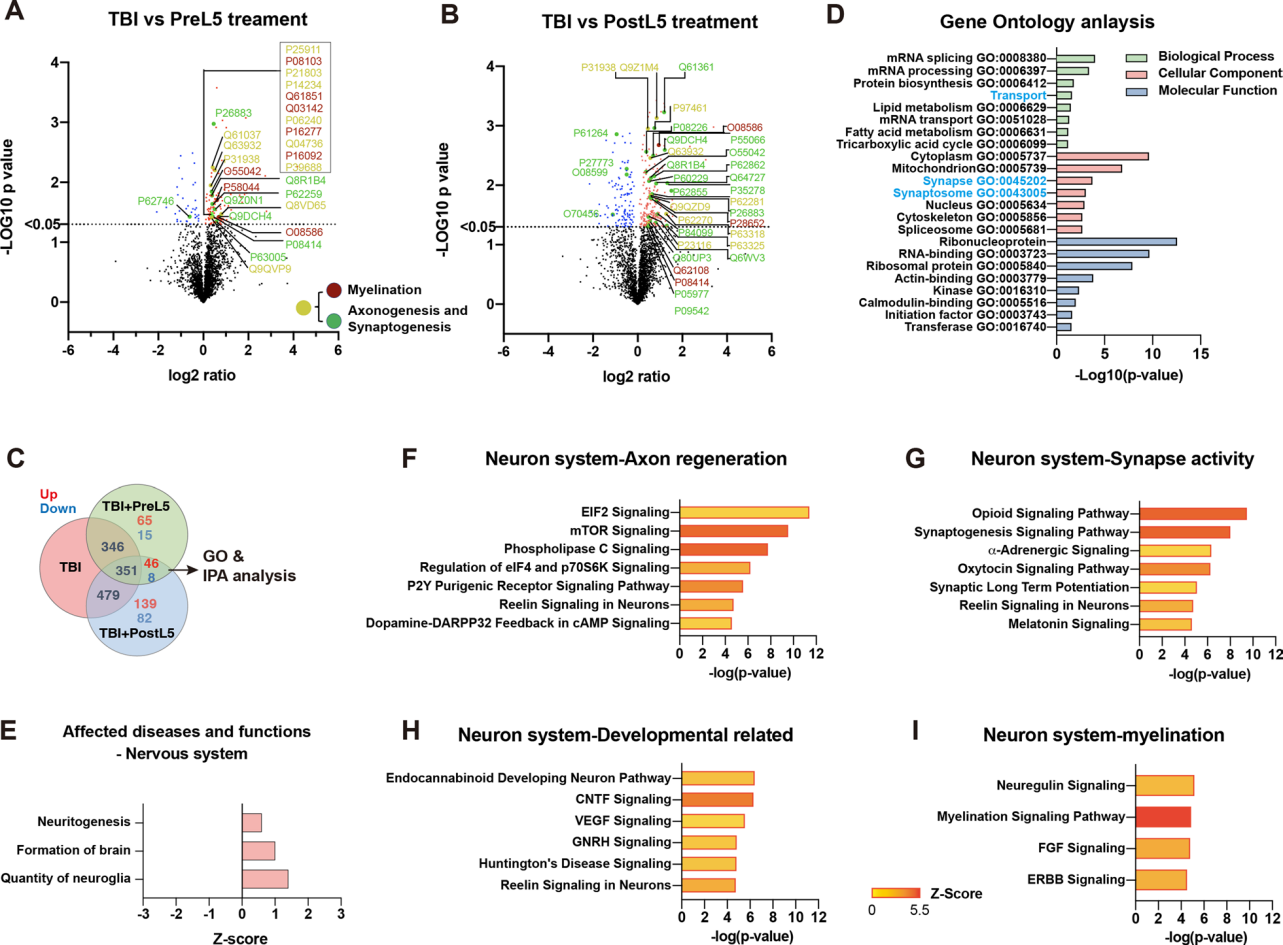
Both pretreatment and post-treatment with CCL5 enhanced neurite and synapse growth and myelination-related signaling pathways in injured cortical tissue.** A** Volcano plot of significant DEPs between mTBI and TBI + CCL5 pretreatment (PreL5) CCL5-KO mouse cortex. **B** Volcano plot of significant DEPs between mTBI and TBI + CCL5 post-treatment (PostL5) CCL5-KO mouse cortex. DEPs: *p*-value < 0.05 in comparison to TBI, respectively. Colored points represent: log2 ratio > 0 upregulated protein (red) and log2 ratio < 0 downregulated protein (blue). Selected axonogenesis, neuritogenesis, synaptogenesis, and myelination pathway-related proteins are highlighted as indicated (Red: myelinations, Green: axonogenesis, neuritogenesis, and synaptogenesis, Yellow: overlapping). **C** Venn diagram comparing DEPs between the TBI group, TBI + CCL5 pretreatment (PreL5), and TBI + CCL5 post-treatment (PostL5) groups. 54 identified proteins (46 up regulated, 8 down regulated) were affected by both treatments. **D** Identified GO terms from each of the three GO groups (Green bar: biological process, Red bar: cellular component, Blue bar: molecular function. Blue character: neuron function-related) were shown. The strength of enrichment of each GO term was indicated by the Log10 p-value (X-axis). **E** IPA analysis identified affected diseases and functions in the nervous system category (Z-score value indicated that functions are predicted to be activated (red). Selected IPA canonical pathways in the nervous system identified significant DEPs related to axon (**F**), synapse (**G**), neuron development (**H**), and myelination (**I**) related signaling pathways in both PreL5 and PostL5 treatments. The Z-score in different identified pathways was shown as a gradient of yellow to red. See also Supplementary Figs. 3, 4, and 5

To further confirm the rescue effect of CCL5 in cortical neuron function, cortical tissues from CCL5-KO mice with TBI, TBI + PreL5, and TBI-PostL5 were harvested and analyzed by LC-MS/MS. GO enrichment evaluated the entire list of 1531 proteins and identified 134 proteins from PreL5 group vs. TBI, 275 proteins from PostL5 vs. TBI, and 54 proteins from PreL5 overlapping with PostL5 vs. TBI (45 up-regulation, 8 down-regulations. adjusted p-value < 0.05) (Fig. 5A–C and Supplementary Figs. 3A, 4 A-B, 5A-B also see Supplementary Data file 1). Identified GO terms for each category in PreL5 or PostL5 treatment are shown in Supplementary Fig. 4C and 5C and Supplementary Data file 6, 7. The overlapping portions of PreL5 and PostL5 were analyzed by DAVID GO analysis and IPA (Fig. 5C). GO analysis identified that CCL5 treatment affected transport, synapse, and synaptosome-related pathways in the cellular component category (Fig. 5D, Blue character—Supplementary Data File 4. CCL5 treatment reduced abdominal lesion inflammation, necrosis neurodegeneration, the organismal death, organ degeneration, and degeneration of brain categories in IPA analysis (Supplementary Fig. 3B). The major affected diseases and functions are cell death and survival, organismal injury and abnormality, especially Nervous system development, and Function and cellular assembly and organization (Supplementary Fig. 3C). In the nervous system category, the quantity of neuroglia, brain formation, and neuritogenesis were increased in the TBI + CCL5 treatment groups (Fig. 5E). The reduced axonogenesis, neuritogenesis, and myelination-related signaling pathways in KO TBI tissue, such as eIF2, mTOR, synaptogenesis, Neuregulin, and myelination signaling were all increased after CCL5 treatments (Fig. 5F–I, and Supplementary Fig. 3D). IPA analysis showed up-regulation in the axon regeneration signaling pathway (Fig. 5F), synaptogenesis signaling pathway (Fig. 5G), and myelination signaling pathway (Fig. 5I) in both PreL5 and PostL5 CCL5 treatment. Interestingly, CCL5 treatment also enhanced neuron development-related signaling, such as CNTF, VEGF, Huntington’s disease, and Reelin (Fig. 5H). (The gradient of yellow to red indicates the Z-score value in Fig. 5F–I.). Proteins identified in each pathway are listed in the Supplementary Data File 5, and their connections in different pathways are in Supplementary Fig. 3F, G.

The upstream analysis identified several proteins correlated with neuron activation (PI3 kinase, PKA, mTOR pathway-related, such as OTUD3, FUNDC2, PPP1R1B, HTR6), neuronal migration (HTR6, DCLK1), retrograde transport (DCLK1), dendrite development and synaptic vesicle transport release (LRP8, SV2C), as well as transcription factors increasing neurogenesis (TCF7L2, HDAC7) or cortical complexity formation (LGALS3BP) (green labeled in Supplementary Fig. 3E). ACSBG1 promotes long-chain fatty acid metabolism and myelinogenesis (red marked in Supplementary Fig. 3E). These proteins and signaling pathways may facilitate the restoration of cortical neuron regrowth after trauma.

#### Cortical neuron axonogenesis, synaptogenesis, and myelination were enhanced by CCL5 treatment

**Fig. 6:**
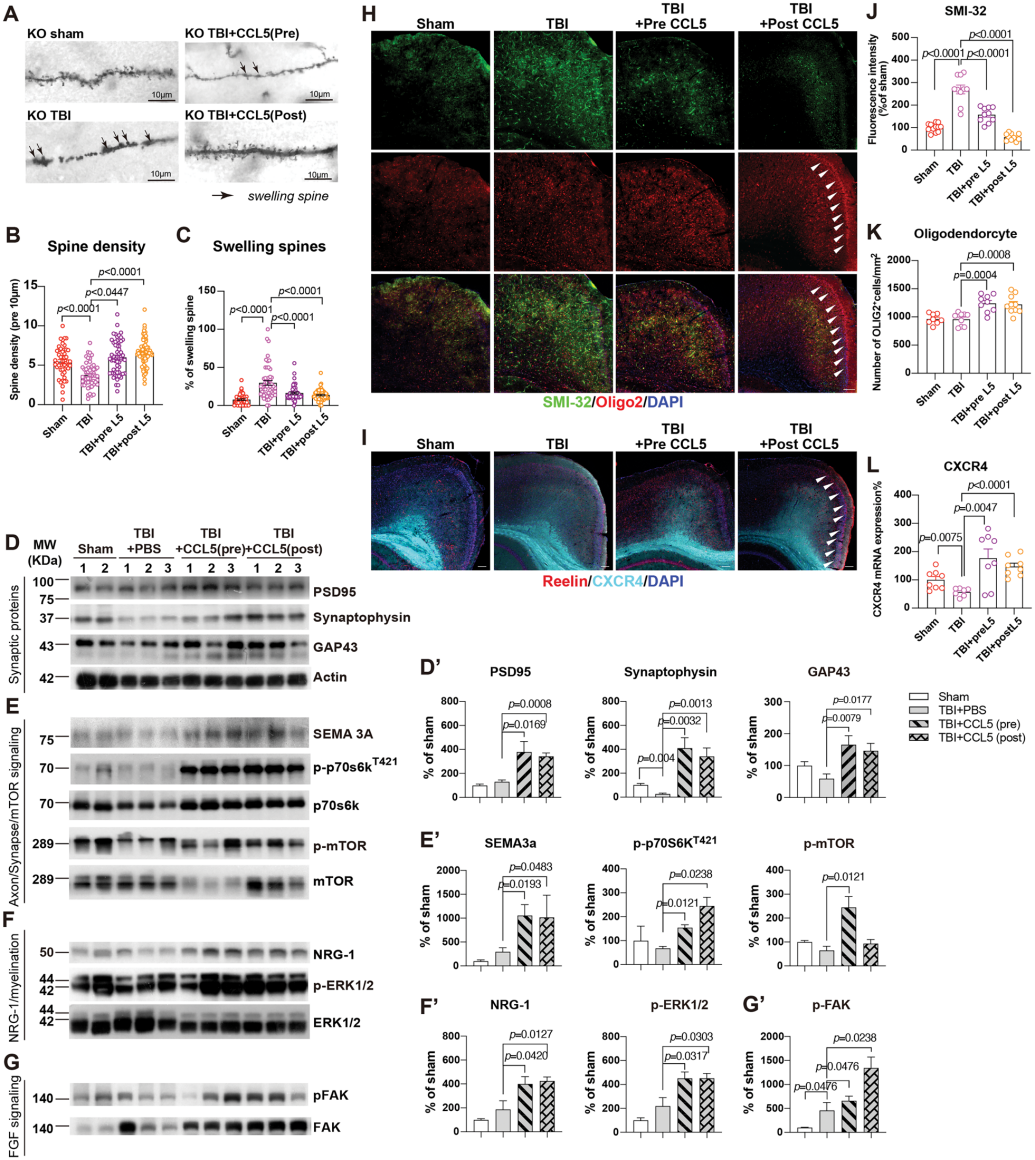
CCL5 treatment enhanced synaptogenesis and myelination by activating the mTOR signaling pathway and the NGR-ERBB signaling pathway after mTBI. Golgi staining of cortical neurons in sham, TBI, TBI with CCL5 pretreatment (PreL5), and TBI with CCL5 post-treatment (PostL5) groups of mice. **A** The representative images of neurites and dendritic spines in different groups of mice. Black arrows point to swollen spines. Scale bar = 10 µm. The spine density (**B**) and the number of swollen spines (**C**) were quantified in different groups of mice (*n* = 10 in each group). (**B**: sham vs TBI, *p* < 0.0001; TBI vs Pre-L5, *p* = 0.0447; TBI vs Post-L5, *p* < 0.0001). (**C**: sham vs TBI, *p* < 0.0001; TBI vs Pre-L5, *p* < 0.0001; TBI vs Post-L5, *p* < 0.0001) **D** The expression of synaptic proteins – PSD95 and synaptophysin in KO mice cortex after mTBI with/without CCL5 administration. (**D’**: PSD95: TBI vs Pre-L5, *p* = 0.0169; TBI vs Post-L5, *p* = 0.0008; Synaptophysin: sham vs TBI, *p* = 0.004; TBI vs Pre-L5, *p* = 0.0032; TBI vs Post-L5, *p* = 0.0013; GAP43: TBI vs Pre-L5, *p* = 0.0079; TBI vs Post-L5, *p* = 0.0177). Western blot analyzed the expression of **E** axon-related signaling proteins - Sema3, EIF2, and mTOR phosphorylation. (**E’**: SEMA3a: TBI vs Pre-L5, *p* = 0.0193; TBI vs Post-L5, *p* = 0.0483; p-p70S6^T421^: TBI vs Pre-L5, *p* = 0.0121; TBI vs Post-L5, *p* = 0.0238; p-mTOR: TBI vs Pre-L5, *p* = 0.0121). **F** myelination-related proteins - Neuregulin, Erk, and SMI32 (**F’**: NRG-1: TBI vs Pre-L5, *p* = 0.0420; TBI vs Post-L5, *p* = 0.0127; p-ERK1/2: TBI vs Pre-L5, *p* = 0.0317; TBI vs Post-L5, *p* = 0.0303); and **G** FGF signaling - FAK phosphorylation (**G’**: p-FAK: sham vs TBI, *p* = 0.0476; TBI vs Pre-L5, *p* = 0.0476; TBI vs Post-L5, *p* = 0.0238) in injured cortex in different groups. (*n* = 4 ~ 5 in each group) **H** The immunostaining of unmyelinated axon - SMI-32 and oligo-2 in 4 groups of CCL5-KO mouse cortex; arrows point to the Oligo-2 positive cells under the pial surface around the injured cortex. The quantification results were in (**J**) SMI-32 and (**K**) oligodendrocytes. (**J**: sham vs TBI, *p* < 0.0001; TBI vs Pre-L5, *p* < 0.0001; TBI vs Post-L5, *p* < 0.0001). (**K**: TBI vs Pre-L5, *p* = 0.0004; TBI vs Post-L5, *p* = 0.0008). **I** The immunostaining of Reelin (red, arrows) and CXCR4 (Cyan) in mouse cortex in different groups of mice; the q-PCR quantitative result of CXCR4 in mouse cortex **L** (**L**: sham vs TBI, *p* = 0.0075; TBI vs Pre-L5, *p* = 0.0047; TBI vs Post-L5, *p* < 0.0001). Data were analyzed by unpaired *t*-test. See also Supplementary Fig. 6

We further validated these findings from proteomic analysis anatomically. Synapse and spine structure in sham, TBI, and TBI with pretreatment of CCL5 (TBI + PreL5) or TBI with post-treatment of CCL5 (TBI + PostL5) cortex were examined using Golgi staining. The reduced spine density in the TBI group of KO mice was restored in the TBI + PreL5 and TBI + PostL5 groups of mice (Fig. 6A-B); the increased number of swollen spines in the TBI group was reduced in both TBI + PreL5 and TBI + PostL5 groups of mice (Fig. 6A, C). Synaptic proteins - PSD95 , synaptophysin and GAP43 in the injured mouse cortex also increased after CCL5 treatment (Fig. 6D, D’).

Those reduced axonal and spine formation-related signaling proteins in the TBI group of CCL5-KO mice, including Sema3A, phosphor-p70S6, and phosphor-mTOR, were increased, in both TBI + preCCL5 and TBI + PostCCL5 mouse cortex (Fig. 6E, E’). Myelination-related signaling pathways proteins—NRG-1, phosphor-Erk (Fig. 6F, F’), and phosphor-FAK-increased (Fig. 6G, G’). Unmyelinated neurofilament labeled by SMI-32 was reduced in those mice receiving CCL5 treatment (Fig. 6H, J) with increased oligodendrocytes (Arrowheads pointed, Fig. 6H, K). In addition, receptor CXCR4, identified by LC-MS/MS, was also increased in the CCL5-administered cortex by both immunostaining and q-PCR analysis (Fig. 6I–L, and Supplementary Fig. 6). Interestingly, Reelin signal was increased in the post-CCL5 treatment group which guide axon extension/migration in the cortex region (Fig. 6I, and Supplementary Fig. 6).

Although neuronal function in WT mice recovered from mTBI quickly (7–14 dpi), the recovery of spine and axon was seen only after 14 dpi. Therefore, similar CCL5 administration was also performed in WT mice. The mNSS, Rotarod, beam walking and ART scores changed significantly in WT mice receiving CCL5 after TBI, especially in the PostL5 group (Supplementary Fig. 7A–C). The spine density of cortical neurons was significantly increased in both pre- and post-CCL5 treatment groups (Supplementary Fig. 7E, F) and the percent of swollen spines was reduced by CCL5 administration at 14 dpi (Supplementary Fig. 7E, G) by Golgi staining. The fluorescent intensity of SMI-32 immunostaining was reduced in both pre- and post-CCL5 treatment groups of mice (Supplementary Fig. 7H, I); the number of Oligo2 positive cells was increased in the Post-CCL5 group (Supplementary Fig. 7H, J).

CCL5 administration significantly increased the protein expression of synaptophysin and GAP43 in the injured mouse cortex in both pre- and post-CCL5 treated groups (Supplementary Fig. 7K, K’), but the protein level of PSD95 was only also slightly increased after CCL5 treatment (Supplementary Fig. 7K, K’). With the axon guidance and mTOR related signaling molecules, Post-CCL5 administration significantly increased Reelin expression as well as p70S6K and mTOR phosphorylation (Supplementary Fig. 7L, L’), but not Sema3a (Supplementary Fig. 7L, L’). Both pre- and post-CCL5 treatment increased the NRG-1 downstream molecule – Erk1/2 phosphorylation (Supplementary Fig. 7M, M’) but not NRG-1. Interestingly, post-CCL5 treatment increased FAK protein expression but not the phosphorylation (Supplementary Fig. 7N, N’). Although CCL5 administration showed some different activations of signaling molecules between WT and CCL5-KO, CCL5 makes a significant contribution to in vivo axonogenesis, synaptogenesis, and remyelination processes after brain injury in both WT and CCL-KO mice.

### CCL5 promoted growth cone formation and sprouting through receptor CCR5 and mTOR and ERK signaling pathway

**Fig. 7:**
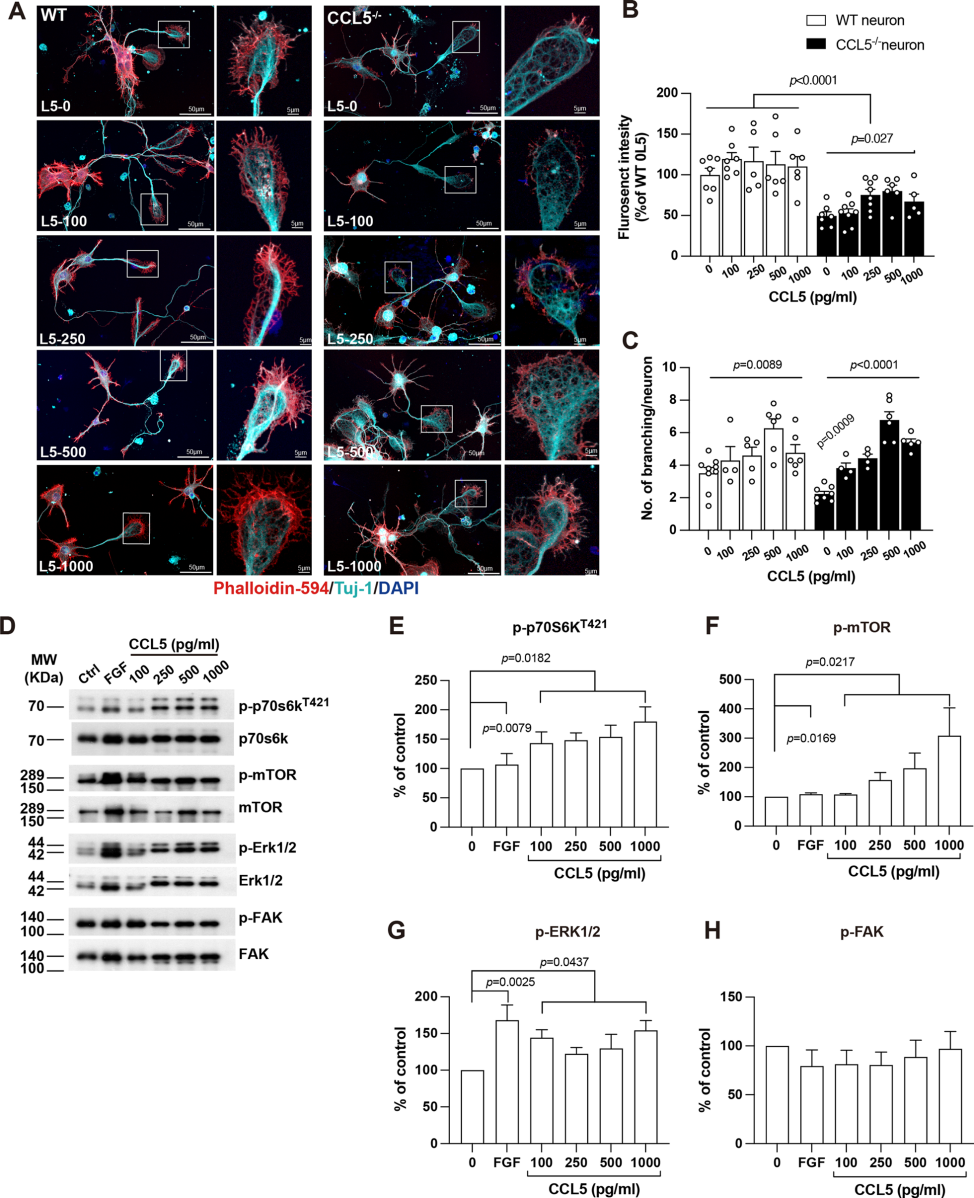
CCL5 increased growth cone formation through activating the mTOR and FAK pathway. **A** Phalloidin (red) labeled the filopodia in axon growth cones of WT or CCL5-KO neurons after treating with CCL5 (0, 100, 250, 500, 1000 pg/ml). Tuj-1 labeled neurites, and DAPI labeled the nucleus. Phalloidin labeled growth cones in different groups were enlarged in the boxed regions: scale bar = 50 μm and 5 μm. The fluorence intensity of Phalloidin and neurite branching in different groups was quantified in **B**,** C**. (**B**: KO neuron treated with CCL5, *p* = 0.027; WT vs. KO neuron, *p* < 0.0001). (**C**: WT neuron treated with CCL5, *p* = 0.0089; KO neuron treated with CCL5, *p* < 0.0001; WT contol vs. KO contol, *p* = 0.0009 by *t*-test). **D** The activation of p70s6k/mTOR, NRG-1, FAK, and Erk signaling proteins in neurons after treatment with CCL5 (0, 100, 250, 500, 1000 pg/ml) in westen blot analysis. FGF was taken as positive control. Quantification data were in **E-H**. **E** The activation of p70s6k by CCL5 *p* = 0.0182; the activation of p70s6k by FGF *p* = 0.0079. **F** The activation of mTOR by CCL5 *p* = 0.0217; the activation of mTOR by FGF *p* = 0.0169. **G** The activation of ERK1/2 by CCL5 *p* = 0.0437; the activation of ERK1/2 by FGF *p* = 0.0025. Data was analyzed by one-way ANOVA in same group and two-way ANOVA between groups. Data between control and FGF treatment was analyzed by *t*-test

CCL5 promotes synaptogenesis in hippocampal neurons through the PI3K-Akt-GSK3β pathway shown in our previous study [1], but the growth cone regrowth is the critical step of axonogenesis after injury. Herein, this study identified several signaling pathways activated by CCL5 in mouse brains, such as the mTOR, Rho, and FGF pathways; we further validated those signaling pathways in in vitro cortical neuron cultures. Cortical neurons were cultured from WT and CCK5-KO mice and treated with different doses of CCL5 (0, 250, 500, and 1000 pg/ml of CCL5). FGF treatment was taking as a positive control. Phalloidin was used to label the growth cones, and Tuj-1 labeled the whole neuron structure. The signal intensity of Phalloidin was significantly lower in the CCL5-KO neurons around growth cones compared to WT neurons, which increased with CCL5 treatment in KO neurons but slightly in WT neurons (Fig. 7A, B). The number of branching was lower in CCL5-KO neurons, and increased with CCL5 treatment in both WT and KO neurons (Fig. 7A, C). The phosphorylation of mTOR/p70s6k and Erk signaling was also increased after CCL5 treatment (Fig. 7D–H).

**Fig. 8:**
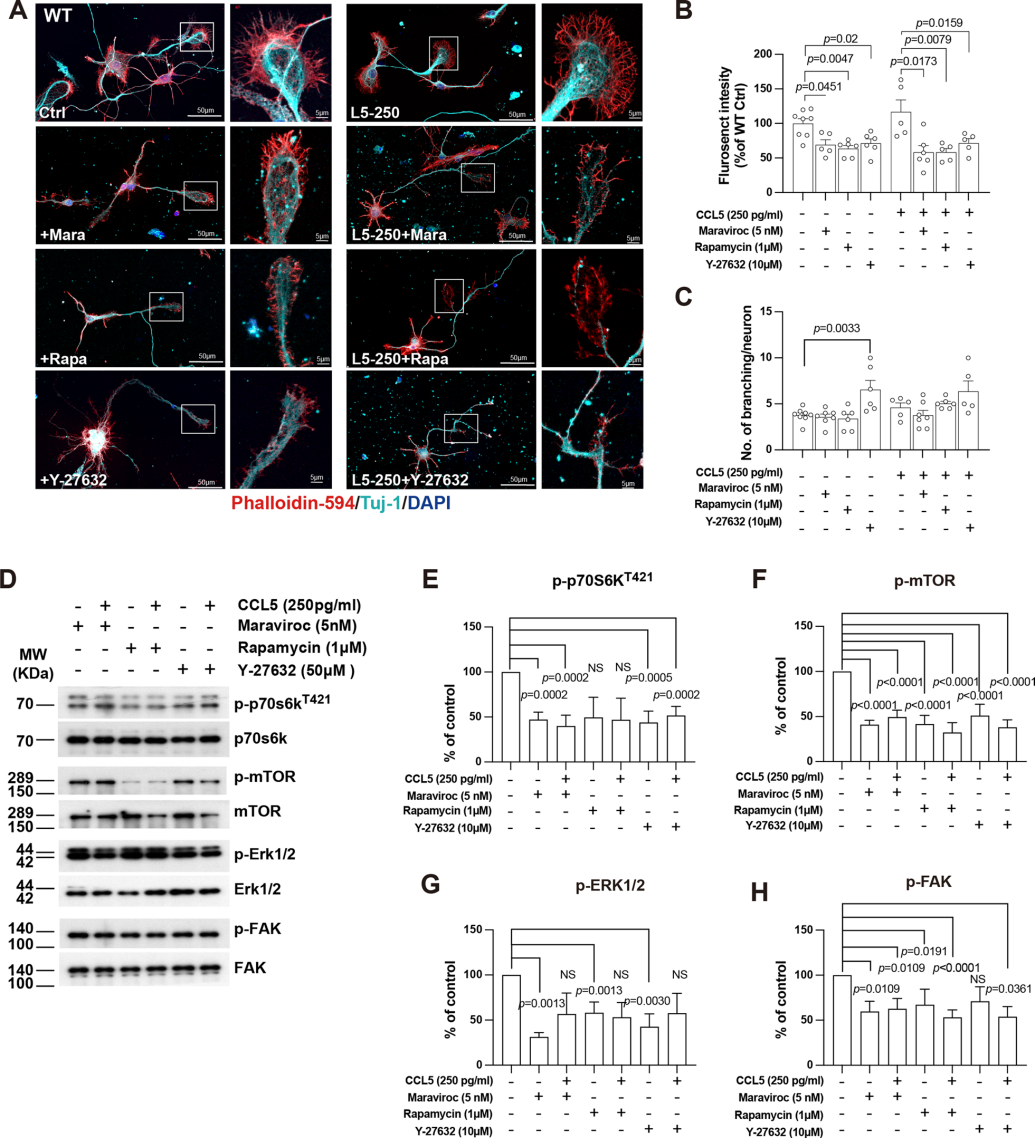
Inhibiting CCR5 receptor and mTOR signaling pathway reduced CCL5’s effect on growth cone formation. **A** Phalloidin (red) labeled the filopodia in axon growth cones of WT neurons treating with CCL5 (250 pg/ml), CCR5 inhibitor-Maraviroc (5 nM), mTOR inhibitor-Rapamycin (1 µM) and ROCK inhibitor-Y27632 (50 µM). Tuj-1 labeled neurites, and DAPI labeled the nucleus. Phalloidin and growth cones in different groups were enlarged in the boxed regions: scale bar = 50 μm and 5 μm. The fluorence intensity of Phalloidin and number of branching in different groups was quantified in **B**,** C**. (**B**: control vs. Maraviroc, *p* = 0.0451; control vs. Rapamycin (Rapa), *p* = 0.0047; control vs. Y-27632, *p* = 0.02. CCL5 vs. CCL5 + Maraviroc, *p* = 0.0173; CCL5 vs. CCL5 + Rapamycin, *p* = 0.0079; CCL5 vs. CCL5 + Y-27632, *p* = 0.0159.). (**C**: control vs. Y-27632, *p* = 0.0033, by *t*-test). **D** The activation of p70s6k/mTOR, NRG-1, FAK, and Erk signaling proteins in neurons after cotreatment with CCL5 (250 pg/ml) and CCR5 inhibitor-Maraviroc, Y27632 or Rapamycin (1 µM) in western blot analysis. FGF was taken as positive control. Quantification data were in **E-H**. **E** The quantification of p70s6k activation. (Control vs. Maraviroc, *p* = 0.0002; control vs. CCL5 + Maraviroc, *p* = 0.0002; control vs. Y-27632, *p* = 0.0005; control vs. CCL5 + Y-27632, *p* = 0.0002. ) **F** The quantification of mTOR activation. (Control vs. Maraviroc, *p* < 0.0001; control vs. CCL5 + Maraviroc, *p* < 0.0001; control vs. Rapa, *p* < 0.0001; control vs. CCL5 + Rapa, *p* < 0.0001; control vs. Y-27632, *p* < 0.0001; control vs. CCL5 + Y-27632, *p* < 0.0001.). **G** The quantification of ERK1/2 activation. (Control vs. Maraviroc, *p* = 0.0013; control vs. Rapa, *p* = 0.0013; control vs. Y-27632, *p* = 0.0030.) **H** The quantification of FAK activation. (Control vs. Maraviroc, *p* < 0.0109; control vs. CCL5 + Maraviroc, *p* = 0.0109; control vs. Rapa, *p* = 0.0191; control vs. CCL5 + Rapa, *p* < 0.0001; control vs. CCL5 + Y-27632, *p* = 0.0361.). Data was analyzed by unpaired *t*-test. NS: no significant difference

Blocking CCL5 receptor - CCR5 with a FAD proved inhibitor - Maraviroc (5 nM) reduced phalloidin signals intensity (Fig. 8A, B) but not branching (Fig. 8A, C); the activation of FAK/Erk1/2, mTOR/p70s6k signaling by CCL5 was also reduced by Maraviroc (Fig. 8D–H). Rapamycin, the inhibitor of mTOR signaling, inhibits the activation of mTOR and caused inactivation of p70s6K (Fig. 8E) which also abolished CCL5’s effect on growth cones (Fig. 8A, B). The Rho kinase inhibitor - Y27632 (50 µM, ROCK inhibitor) blocked CCL5’s effect on growth cone (Fig. 8A, B) but increased the branching number (Fig. 8C) which modulated the activation of Erk/FAK singlaing upon CCL5 administration. Thus, the CCL5/CCR5 axis promotes cortical neuron axon formation through mTOR and Erk signaling pathways.

## Discussion

The present study identifies a unique role of the chemokine CCL5 in axonal regrowth after brain trauma. Our data also specifically reveal that CCL5, by promoting axonogenesis and remyelination, restores cortical neuron structure and function after injury. Intranasal administration of CCL5 into the brain after injury at either the acute phase (single dosage of CCL5, 300 pg/g) or the chronic phase (low dose of CCL5, 30 pg/g every two days) significantly augments animal neurological behavioral performance and also neuronal structural reconstruction.

Most patients recover from mild brain injury around 7 days and show no significant neurological impairment in the clinic; however, the neuronal structure and connections require weeks to months to repair after injury [13]. Repairing neurites and reconnection between neurons during the chronic/remodeling phase are critical for preventing neurodegeneration after brain injury. Herein, the motor and sensory function improved in WT mice after 1–2 weeks (Fig. 1D, F), but the structure of axons, neurites, and dendritic spine started to recover from 14 dpi to 28 dpi (Fig. 2A–E). Interestingly, the recovery of neurological sores was reduced in CCL5-KO mice with reduced neurites and spine structures (Fig. 2). This supports the critical role of CCL5 in the chronic/remodeling phase after TBI. Pretreatment with CCL5 to mimic endogenous CCL5 effects in the brain and post-treatment with CCL5 was used to test the possibility of using CCL5 in TBI patients. We have previously identified that pretreatment with CCL5 activates GPX1 expression, reduces tissue oxidative stress, reduces memory impairment, and protects hippocampal neurons after injury. Herein, we also show the reduction of oxidative stress in cortex by CCL5 pretreatment (Supplementary Fig. 4D, purple-Free radical scavenging) but not in the post-treatment protocol (Supplementary Fig. 5D). Importantly, treating mice with CCL5, both with pre- and post-treatments, affected mainly neuron function, such as enhanced mTOR and Erk/FGF-mediated axonogenesis and synaptogenesis signaling in our animal in vivo (Fig. 6) and in the in vitro culture models (Fig. 7). mTOR signaling is a novel therapeutic strategy for TBIs, which may promote axon regeneration in the adult CNS [10, 45].

CCL5 binds to many receptors in the brain; inhibition of CCR5, one of CCL5’s receptors, benefits recovery after a stroke or TBI in mice [26]. CCR5 activation promotes NLRP1-dependent neuronal pyroptosis after intracerebral hemorrhage [58]; however, our study identified a reduction of necrosis, neurodegeneration, organ degeneration, and degeneration of the brain in TBI mice treated with CCL5 (Supplementary Fig. 3B). Most of those studies treated mice with the CCR5 inhibitor—Maraviroc in the acute phase, such as 0–5 days in a CCI model or a moderate TBI model [17, 26, 36] which is very different from our mild close-head injury model. CCI is an open head injury model which causes brain tissue damage or even tissue lost and activates a severe inflammatory response. Inhibiting CCR5 mediated inflammation in the acute phase can successfully reduce inflammation and also balance chemokine and cytokine levels and responses after these brain traumas. However, there is no study using Maraviroc in the chronic phase. Our model is closer to normal close head mild traumatic brain injury conditions which do not cause severe inflammation and no open wound. Importantly, we treated animals after 3 dpi with low doses of CCL5 (30pg/g) every two days until 28 dpi which improved not only motor and sensory functions but also spine structures and remyelination (reduced SMI32 and increased Oligo2) in both WT and CCL5-KO mouse cortical injury regions. In addition, CCR5 is the critical receptor in CCL5, promoting growth cone formation and mTOR signaling activation in the primary neuron culture system (Fig. 8). In a previous study, CCL5 has been identified as the downstream key factor of CNTF. It promotes optic nerve regeneration through the CCR5 receptor on RGCs (retina ganglia cells) [56]. Taken together, our findings show that the CCL5/CCR5 axis can also promote cortical nerve regeneration during TBI chronic phase.

Administration of CCL5 activates myelination and axon guidance-related signaling (Fig. 5H). Oligodendrocytes are critical for synapse and axon pruning processes [14]; however, the role of CCL5 in oligodendrocytes is largely unclear. Lanfranco et. al. using RNAscope, showed that CCL5 is highly expressed in Nkx2.2 positive oligodendrocytes adjacent to the corpus callosum [30] and CCL5 promotes oligodendrocytes precursor proliferation in an oligodendrocyte cell line [27]. We identified more unmyelinated neurofilaments (SMI32 positive) in CCL5 deficient mice (Fig. 3K–L) and showed that CCL5 treatment reduced SMI32 positive signaling and increased Oligo2 signaling in CCL5 deficient mice after TBI (Fig. 6H, I). In particular, this is important for Oligo2 positive cells aligned under the cortex (Fig. 6H); these are Reelin positive - Cajal-Retzius cells, which function for axon guidance and migration, as well as brain structure development. In addition, NRG1/ErbB signaling also participates in oligodendrocyte survival and proliferation (Supplementary Fig. 3G). Altogether, our data suggest a strong effect of CCL5 in in vivo oligodendrocyte related remyelination process after axon injury. This augments the possibility of using CCL5 as a therapeutic stratagem.

CCL5 treatment also influences development-related signaling, such as CNTF, VEGF, GNRH, and Huntington’s disease-related signaling molecules (Fig. 5H). A co-receptor-CXC chemokine receptor 4 (CXCR4) increased in CCL5 treated tissue groups in our proteomic analysis but not CCR5 (Supplementary Data file 5). Quantitative PCR and immunostaining showed the increased expression of CXCR4 in cortical tissue after CCL5 administration (Fig. 6I, L). CXCR4 has marked activity in cell proliferation and tissue regeneration [4]; CXCR4 also regulates neuronal migration and axonal pathway finding [50], CXCR4 signaling defines the initial trajectory of mammalian motor axons [34], especially the CXCR4/CXCL12-mediated entrapment of axons at the injury site in optic nerve regeneration [21]. Synaptically released CCL5 triggers CXCR4 surface expression and enhances the cellular response to CXCL12 in a neuronal cell line study [16]. CXCR4 is expressed along the ventricular system, in the olfactory bulb, and within the subgranular zone of the dentate gyrus [52]; intranasal delivery of CCL5 through the rostral migratory stream (RMS) thus allows CCL5 to have a greater chance to bind to and activate CXCR4-related signaling.

The impact on cortical neurons and hippocampus neurons were very different after weight-drop induced brain injury from our study. First, we noticed a higher oxidative stress (Hydroxyprobe positive) in hippocampus neurons after mild TBI but lower levels in the cortex region in the same experiments; oxidative stress in cortex is less severe than in hippocampus (Oxidative stress in cortex related data is not been published; hippocampus related data was published in our previous paper [22]). CCL5 activation of the antioxidant GPX-1 in the acute phase (within 24 h) to reduce the oxidative stress is more important for hippocampal neuron and memory recovery. Second, cortex tissue received more structural deformation than hippocampus and also axon elastration with a weight-drop model. Therefore, axon repair and remyelination processes are important for cortical neurons. This repair system is a chronic process taking place around 14–28 dpi. Low dose (30 pg/g) multiple time administration of CCL5 after mTBI show stronger effects on axon and synapse regrowth processes and remyelination, especially in WT mice (Fig. 6H, J, K, Supplementary Fig. 7H–J). CCL5 also activates Reelin signaling to guide the regrowth process (Fig. 6H, Supplementary Fig. 7H, L). Reduced spine number and synaptic protein—synaptophysin and GAP43 after injury increased by CCL5 administration (Fig. 6A–C, Supplementary Fig. 7G, K). CCL5 activates mTOR and Erk signaling to facilitate axon regrowth (growth cone formation) and remyelination process. Our finding provides an important mechanism of CCL5 to the finding of raised expression of CCL5 around the axonal transection side in mice after injury [3] and also the possible stratagem of using CCL5 and its antagonist—Maraviroc in TBI at different disease phase/timing.

## Conclusions

In conclusion, we identified that CCL5 shows two functions to promote neuronal restoration after brain injury. First, increasing CCL5 around the axonal transection site in the mouse brain after injury activates the mTOR signaling pathway to facilitate growth cone formation, axon regrowth, synapse formation, and cell migration; second, CCL5 activates NRG-1/ErbB signaling to promote oligodendrocyte remyelination and axon sprouting. Together, these two factors facilitate axonal regeneration after axonal injury to restore cortex neuron function.

## Supplementary Information

Supplementary Material 1.

Supplementary Material 2.

Supplementary Material 3.

Supplementary Material 4.

Supplementary Material 5.

Supplementary Material 6.

Supplementary Material 7.

Supplementary Material 8.

## Data Availability

Data are available from the corresponding authors upon reasonable request.

## Abbreviations

ACSBG1: Acyl-CoA Synthetase Bubblegum Family Member 1
Akt: Protein kinase B
ART: Adhesive removal tests
ATP: Adenosine triphosphate
BDNF: Brain-Derived Neurotrophic Factor
CCL5/RANTES: C-C Motif Chemokine Ligand 5/ (regulated on activation, normal T cell expressed and secreted
CCL5-KO: CCL5 knockout
CCR5: C-C chemokine receptor type 5
CDK5: Cyclin-dependent kinase 5
CNS: Central Nervous System
CNTF: Ciliary neurotrophic factor
CTE: Chronic Traumatic Encephalopathy
CTX: Cortex
CXCR4: Chemokine (C-X-C motif) receptor 4
DAI: Diffuse axonal idiopathic injury
DAVID: The Database for Annotation, Visualization and Integrated Discovery
DCLK1: Doublecortin like kinase protein 1
EGF: Epidermal growth factor
EIF2: eukaryotic Initiation Factor 2
eIF4: eukaryotic Initiation Factor 4
ELISA: Enzyme-linked immunosorbent assay
ErbB: erythroblastic oncogene B
Erk: Extracellular signal-regulated kinase
FAK: Focal adhesion kinase
FGF: fibroblast growth factor
FUNDC2: FUN14 domain-containing protein 2
GCS: Glasgow Coma Scale
GFAP: Glial Fibrillary Acidic Protein
GO: Gene Ontology
GPX1: glutathione peroxidase 1
GSK3: Glycogen synthase kinase 3
HDAC7: Histone deacetylase 7
HTR6: 5-Hydroxytryptamine Receptor 6
i.n.: Intranasal
Iba1: Ionized calcium-binding adapter molecule 1
IPA: Ingenuity Pathway Analysis
LC-MS/MS: Liquid Chromatography-mass spectrometry/ mass spectrometry
LGALS3BP: lectin galactoside-binding soluble 3 binding protein
LRP8: Low-density lipoprotein receptor-related protein 8
MAPK: Mitogen-activated protein kinase
mNSS: Modified neurological severity score
mTOR: Mammalian target of rapamycin
NLRP1: NLR Family Pyrin Domain Containing 1
NRG-1: Neuregulin 1
OTUD3: OTU Deubiquitinase 3
P70s6k: Ribosomal protein S6 kinase beta-1
PFC: Prefrontal Cortex
PI3K: Phosphoinositide 3-kinases
PKA: Protein kinase A
PPP1R1B: Protein phosphatase 1 regulatory subunit 1B
PSD95: Postsynaptic density protein 95
Rac-1: Ras-related C3 botulinum toxin substrate 1
RhoA: Ras homolog family member A
Sema3A: Semaphorin 3A
SV2C: Synaptic Vesicle Glycoprotein 2C
TAI: Traumatic axonal injury
TBI: Traumatic brain injury
TCF7L2: Transcription Factor 7 Like 2
VEGF: Vascular endothelial growth factor
WT: Wildtype

## References

[1] Ajoy R, Lo YC, Ho MH, Chen YY, Wang Y, Chen YH, Jing-Yuan C, Changou CA, Hsiung YC, Chen HM, Chang TH, Lee CY, Chiang YH, Chang WC, Hoffer B, Chou SY. CCL5 promotion of bioenergy metabolism is crucial for hippocampal synapse complex and memory formation. Mol Psychiatry. 2021;26(11):6451–68. 33931731 10.1038/s41380-021-01103-3 PMC8760051

[2] Albert V, Subramanian A, Agrawal D, Bhoi SK, Pallavi P, Mukhopadhayay AK. RANTES levels in peripheral blood, CSF and contused brain tissue as a marker for outcome in traumatic brain injury (TBI) patients. BMC Res Notes. 2017;10(1):139. 28340601 10.1186/s13104-017-2459-2 PMC5366123

[3] Babcock AA, Kuziel WA, Rivest S, Owens T. Chemokine expression by glial cells directs leukocytes to sites of axonal injury in the CNS. J Neurosci. 2003;23(21):7922–30. 12944523 10.1523/JNEUROSCI.23-21-07922.2003 PMC6740601

[4] Bianchi ME, Mezzapelle R. The chemokine receptor CXCR4 in cell proliferation and tissue regeneration. Front Immunol. 2020;11:2109. 32983169 10.3389/fimmu.2020.02109 PMC7484992

[5] Bodnar CN, Roberts KN, Higgins EK, Bachstetter AD. A systematic review of closed Head Injury models of mild traumatic brain Injury in mice and rats. J Neurotrauma. 2019;36(11):1683–706. 30661454 10.1089/neu.2018.6127 PMC6555186

[6] Bouet V, Boulouard M, Toutain J, Divoux D, Bernaudin M, Schumann-Bard P, Freret T. The adhesive removal test: a sensitive method to assess sensorimotor deficits in mice. Nat Protoc. 2009;4(10):1560–4. 19798088 10.1038/nprot.2009.125

[7] Bruggeman GF, Haitsma IK, Dirven CMF, Volovici V. Traumatic axonal injury (TAI): definitions, pathophysiology and imaging—a narrative review. Acta Neurochir (Wien). 2021;163(1):31–44. 33006648 10.1007/s00701-020-04594-1 PMC7778615

[8] Chou SY, Weng JY, Lai HL, Liao F, Sun SH, Tu PH, Dickson DW, Chern Y. Expanded-polyglutamine huntingtin protein suppresses the secretion and production of a chemokine (CCL5/RANTES) by astrocytes. J Neurosci. 2008;28(13):3277–90. 18367595 10.1523/JNEUROSCI.0116-08.2008 PMC6670608

[9] Cooke P, Janowitz H, Dougherty SE. Neuronal Redevelopment and the Regeneration of Neuromodulatory Axons in the adult mammalian Central Nervous System. Front Cell Neurosci. 2022;16:872501. 35530177 10.3389/fncel.2022.872501 PMC9074815

[10] Don AS, Tsang CK, Kazdoba TM, D’Arcangelo G, Young W, Zheng XF. Targeting mTOR as a novel therapeutic strategy for traumatic CNS injuries. Drug Discov Today. 2012;17(15–16):861–8. 22569182 10.1016/j.drudis.2012.04.010 PMC3411916

[11] Dubal DB, Wise PM. Estrogen and neuroprotection: from clinical observations to molecular mechanisms. Dialogues Clin Neurosci. 2002;4(2):149–61. 22034440 10.31887/DCNS.2002.4.2/ddubal PMC3181675

[12] Duncan KA. Estrogen formation and inactivation following TBI: what we know and where we could go. Front Endocrinol (Lausanne). 2020;11:345. 32547495 10.3389/fendo.2020.00345 PMC7272601

[13] Egawa N, Lok J, Washida K, Arai K. Mechanisms of axonal damage and repair after Central Nervous System Injury. Transl Stroke Res. 2017;8(1):14–21. 27566737 10.1007/s12975-016-0495-1 PMC5243173

[14] Fang LP, Bai X. Oligodendrocyte precursor cells: the multitaskers in the brain. Pflugers Arch. 2023;475(9):1035–44. 37401986 10.1007/s00424-023-02837-5 PMC10409806

[15] Filbin MT. Myelin-associated inhibitors of axonal regeneration in the adult mammalian CNS. Nat Rev Neurosci. 2003;4(9):703–13. 12951563 10.1038/nrn1195

[16] Franciszkiewicz K, Boutet M, Gauthier L, Vergnon I, Peeters K, Duc O, Besse B, de Saint Basile G, Chouaib S, Mami-Chouaib F. Synaptic release of CCL5 storage vesicles triggers CXCR4 surface expression promoting CTL migration in response to CXCL12. J Immunol. 2014;193(10):4952–61. 25305322 10.4049/jimmunol.1401184

[17] Friedman-Levi Y, Liraz-Zaltsman S, Shemesh C, Rosenblatt K, Kesner EL, Gincberg G, Carmichael ST, Silva AJ, Shohami E. Pharmacological blockers of CCR5 and CXCR4 improve recovery after traumatic brain injury. Exp Neurol. 2021;338:113604. 33453212 10.1016/j.expneurol.2021.113604

[18] Gardner RC, Byers AL, Barnes DE, Li Y, Boscardin J, Yaffe K. Mild TBI and risk of Parkinson disease: a chronic effects of Neurotrauma Consortium Study. Neurology. 2018;90(20):e1771–9. 29669907 10.1212/WNL.0000000000005522 PMC5957305

[19] Goldstein LE, Fisher AM, Tagge CA, Zhang XL, Velisek L, Sullivan JA, Upreti C, Kracht JM, Ericsson M, Wojnarowicz MW, Goletiani CJ, Maglakelidze GM, Casey N, Moncaster JA, Minaeva O, Moir RD, Nowinski CJ, Stern RA, Cantu RC, Geiling J, Blusztajn JK, Wolozin BL, Ikezu T, Stein TD, Budson AE, Kowall NW, Chargin D, Sharon A, Saman S, Hall GF, Moss WC, Cleveland RO, Tanzi RE, Stanton PK, McKee AC. Chronic traumatic encephalopathy in blast-exposed military veterans and a blast neurotrauma mouse model. Sci Transl Med. 2012;4(134):134ra160. 10.1126/scitranslmed.3003716 PMC3739428 22593173

[20] Gyoneva S, Ransohoff RM. Inflammatory reaction after traumatic brain injury: therapeutic potential of targeting cell-cell communication by chemokines. Trends Pharmacol Sci. 2015;36(7):471–80. 25979813 10.1016/j.tips.2015.04.003 PMC4485943

[21] Hilla AM, Baehr A, Leibinger M, Andreadaki A, Fischer D. CXCR4/CXCL12-mediated entrapment of axons at the injury site compromises optic nerve regeneration. Proc Natl Acad Sci U S A 118(21), 2021. 10.1073/pnas.2016409118 PMC8166183 34011605

[22] Ho MH, Yen CH, Hsieh TH, Kao TJ, Chiu JY, Chiang YH, Hoffer BJ, Chang WC, Chou SY. CCL5 via GPX1 activation protects hippocampal memory function after mild traumatic brain injury. Redox Biol. 2021;46:102067. 34315111 10.1016/j.redox.2021.102067 PMC8327355

[23] Hyder AA, Wunderlich CA, Puvanachandra P, Gururaj G, Kobusingye OC. The impact of traumatic brain injuries: a global perspective. NeuroRehabilitation. 2007;22(5):341–53. 18162698

[24] Ignatov A, Robert J, Gregory-Evans C, Schaller HC. RANTES stimulates Ca2 + mobilization and inositol trisphosphate (IP3) formation in cells transfected with G protein-coupled receptor 75. Br J Pharmacol. 2006;149(5):490–7. 17001303 10.1038/sj.bjp.0706909 PMC2014681

[25] Johnson VE, Stewart W, Smith DH. Widespread tau and amyloid-beta pathology many years after a single traumatic brain injury in humans. Brain Pathol. 2012;22(2):142–9. 21714827 10.1111/j.1750-3639.2011.00513.x PMC3979351

[26] Joy MT, Ben Assayag E, Shabashov-Stone D, Liraz-Zaltsman S, Mazzitelli J, Arenas M, Abduljawad N, Kliper E, Korczyn AD, Thareja NS, Kesner EL, Zhou M, Huang S, Silva TK, Katz N, Bornstein NM, Silva AJ, Shohami E, Carmichael ST. CCR5 is a therapeutic target for recovery after stroke and traumatic brain Injury. Cell 176(5):1143–57 e1113, 2019. 10.1016/j.cell.2019.01.044 PMC7259116 30794775

[27] Kadi L, Selvaraju R, de Lys P, Proudfoot AE, Wells TN, Boschert U. Differential effects of chemokines on oligodendrocyte precursor proliferation and myelin formation in vitro. J Neuroimmunol. 2006;174(1–2):133–46. 16574247 10.1016/j.jneuroim.2006.01.011

[28] Kester MI, van der Flier WM, Visser A, Blankenstein MA, Scheltens P, Oudejans CB. Decreased mRNA expression of CCL5 [RANTES] in Alzheimer’s disease blood samples. Clin Chem Lab Med. 2012;50(1):61–5. 10.1515/CCLM.2011.731 21942811

[29] Koelsch A, Feng Y, Fink DJ, Mata M. Transgene-mediated GDNF expression enhances synaptic connectivity and GABA transmission to improve functional outcome after spinal cord contusion. J Neurochem. 2010;113(1):143–52. 20132484 10.1111/j.1471-4159.2010.06593.x PMC3101255

[30] Lanfranco MF, Mocchetti I, Burns MP, Villapol S. Glial- and neuronal-specific expression of CCL5 mRNA in the rat brain. Front Neuroanat. 2017;11:137. 29375328 10.3389/fnana.2017.00137 PMC5770405

[31] Langlois JA, Rutland-Brown W, Wald MM. The epidemiology and impact of traumatic brain injury: a brief overview. J Head Trauma Rehabil. 2006;21(5):375–8. 16983222 10.1097/00001199-200609000-00001

[32] Lee JK, Schuchman EH, Jin HK, Bae JS. Soluble CCL5 derived from bone marrow-derived mesenchymal stem cells and activated by amyloid beta ameliorates Alzheimer’s disease in mice by recruiting bone marrow-induced microglia immune responses. Stem Cells. 2012;30(7):1544–55. 22570192 10.1002/stem.1125

[33] Lee Y, Morrison BM, Li Y, Lengacher S, Farah MH, Hoffman PN, Liu Y, Tsingalia A, Jin L, Zhang PW, Pellerin L, Magistretti PJ, Rothstein JD. Oligodendroglia metabolically support axons and contribute to neurodegeneration. Nature. 2012;487(7408):443–8. 22801498 10.1038/nature11314 PMC3408792

[34] Lieberam I, Agalliu D, Nagasawa T, Ericson J, Jessell TM. A Cxcl12-CXCR4 chemokine signaling pathway defines the initial trajectory of mammalian motor axons. Neuron. 2005;47(5):667–79. 16129397 10.1016/j.neuron.2005.08.011

[35] Liu K, Tedeschi A, Park KK, He Z. Neuronal intrinsic mechanisms of axon regeneration. Annu Rev Neurosci. 2011;34:131–52. 21438684 10.1146/annurev-neuro-061010-113723

[36] Liu XL, Sun DD, Zheng MT, Li XT, Niu HH, Zhang L, Zhou ZW, Rong HT, Wang Y, Wang JW, Yang GL, Liu X, Chen FL, Zhou Y, Zhang S, Zhang JN. Maraviroc promotes recovery from traumatic brain injury in mice by suppression of neuroinflammation and activation of neurotoxic reactive astrocytes. Neural Regen Res. 2023;18(1):141–9. 35799534 10.4103/1673-5374.344829 PMC9241405

[37] Lucke-Wold BP, Turner RC, Logsdon AF, Bailes JE, Huber JD, Rosen CL. Linking traumatic brain injury to chronic traumatic encephalopathy: identification of potential mechanisms leading to neurofibrillary tangle development. J Neurotrauma. 2014;31(13):1129–38. 24499307 10.1089/neu.2013.3303 PMC4089022

[38] Luo L. Rho GTPases in neuronal morphogenesis. Nat Rev Neurosci. 2000;1(3):173–80. 11257905 10.1038/35044547

[39] Luong TN, Carlisle HJ, Southwell A, Patterson PH. Assessment of motor balance and coordination in mice using the balance beam. J Vis Exp (49), 2011. 10.3791/2376 PMC3197288 21445033

[40] Mouzon B, Chaytow H, Crynen G, Bachmeier C, Stewart J, Mullan M, Stewart W, Crawford F. Repetitive mild traumatic brain injury in a mouse model produces learning and memory deficits accompanied by histological changes. J Neurotrauma. 2012;29(18):2761–73. 22900595 10.1089/neu.2012.2498 PMC13588329

[41] Moye LS, Novack ML, Tipton AF, Krishnan H, Pandey SC, Pradhan AA. The development of a mouse model of mTBI-induced post-traumatic migraine, and identification of the delta opioid receptor as a novel therapeutic target. Cephalalgia. 2019;39(1):77–90. 29771142 10.1177/0333102418777507 PMC6472897

[42] Mychasiuk R, Farran A, Angoa-Perez M, Briggs D, Kuhn D, Esser M. J. A novel model of mild traumatic brain injury for juvenile rats. J Vis Exp (94), 2014. 10.3791/51820 PMC4396946 25548960

[43] Nudo RJ. Recovery after brain injury: mechanisms and principles. Front Hum Neurosci. 2013;7:887. 24399951 10.3389/fnhum.2013.00887 PMC3870954

[44] Omalu B, Bailes J, Hamilton RL, Kamboh MI, Hammers J, Case M, Fitzsimmons R. Emerging histomorphologic phenotypes of chronic traumatic encephalopathy in American athletes. Neurosurgery 69(1):173–83; discussion 183, 2011. 10.1227/NEU.0b013e318212bc7b 21358359

[45] Park KK, Liu K, Hu Y, Smith PD, Wang C, Cai B, Xu B, Connolly L, Kramvis I, Sahin M, He Z. Promoting axon regeneration in the adult CNS by modulation of the PTEN/mTOR pathway. Science. 2008;322(5903):963–6. 18988856 10.1126/science.1161566 PMC2652400

[46] Poplawski GHD, Kawaguchi R, Van Niekerk E, Lu P, Mehta N, Canete P, Lie R, Dragatsis I, Meves JM, Zheng B, Coppola G. And Tuszynski M.H. Injured adult neurons regress to an embryonic transcriptional growth state. Nature. 2020;581(7806):77–82. 32376949 10.1038/s41586-020-2200-5

[47] Raghava N, Das BC, Ray SK. Neuroprotective effects of estrogen in CNS injuries: insights from animal models. Neurosci Neuroecon. 2017;6:15–29. 28845391 10.2147/NAN.S105134 PMC5567743

[48] Schaffert J, LoBue C, White CL, Chiang HS, Didehbani N, Lacritz L, Rossetti H, Dieppa M, Hart J, Cullum CM. Traumatic brain injury history is associated with an earlier age of dementia onset in autopsy-confirmed Alzheimer’s disease. Neuropsychology. 2018;32(4):410–6. 29389151 10.1037/neu0000423 PMC5975092

[49] Simpson IA, Carruthers A, Vannucci SJ. Supply and demand in cerebral energy metabolism: the role of nutrient transporters. J Cereb Blood Flow Metab. 2007;27(11):1766–91. 17579656 10.1038/sj.jcbfm.9600521 PMC2094104

[50] Stumm R, Hollt V. CXC chemokine receptor 4 regulates neuronal migration and axonal pathfinding in the developing nervous system: implications for neuronal regeneration in the adult brain. J Mol Endocrinol. 2007;38(3):377–82. 17339400 10.1677/JME-06-0032

[51] Tokami H, Ago T, Sugimori H, Kuroda J, Awano H, Suzuki K, Kiyohara Y, Kamouchi M, Kitazono T, Investigators R. RANTES has a potential to play a neuroprotective role in an autocrine/paracrine manner after ischemic stroke. Brain Res. 2013;1517:122–32. 23602964 10.1016/j.brainres.2013.04.022

[52] Tran PB, Banisadr G, Ren D, Chenn A, Miller RJ. Chemokine receptor expression by neural progenitor cells in neurogenic regions of mouse brain. J Comp Neurol. 2007;500(6):1007–33. 17183554 10.1002/cne.21229 PMC2758702

[53] Tripathy D, Thirumangalakudi L, Grammas P. RANTES upregulation in the Alzheimer’s disease brain: a possible neuroprotective role. Neurobiol Aging. 2010;31(1):8–16. 18440671 10.1016/j.neurobiolaging.2008.03.009 PMC2803489

[54] Tucker LB, Fu AH, McCabe JT. Performance of male and female C57BL/6J mice on motor and cognitive tasks commonly used in Pre-clinical traumatic brain Injury Research. J Neurotrauma. 2016;33(9):880–94. 25951234 10.1089/neu.2015.3977 PMC4860656

[55] Wang J, Hou Y, Zhang L, Liu M, Zhao J, Zhang Z, Ma Y, Hou W. Estrogen attenuates traumatic Brain Injury by inhibiting the activation of Microglia and astrocyte-mediated neuroinflammatory responses. Mol Neurobiol. 2021;58(3):1052–61. 33085047 10.1007/s12035-020-02171-2

[56] Xie L, Yin Y, Benowitz L. Chemokine CCL5 promotes robust optic nerve regeneration and mediates many of the effects of CNTF gene therapy. Proc Natl Acad Sci U S A. 2021; 118(9). 10.1073/pnas.2017282118 PMC7936361 33627402

[57] Yagura K, Ohtaki H, Tsumuraya T, Sato A, Miyamoto K, Kawada N, Suzuki K, Nakamura M, Kanzaki K, Dohi K, Izumizaki M, Hiraizumi Y, Honda K. The enhancement of CCL2 and CCL5 by human bone marrow-derived mesenchymal stem/stromal cells might contribute to inflammatory suppression and axonal extension after spinal cord injury. PLoS ONE. 2020;15(3):e0230080. 32155215 10.1371/journal.pone.0230080 PMC7064230

[58] Yan J, Xu W, Lenahan C, Huang L, Wen J, Li G, Hu X, Zheng W, Zhang JH, Tang J. CCR5 activation promotes NLRP1-Dependent neuronal pyroptosis via CCR5/PKA/CREB pathway after Intracerebral Hemorrhage. Stroke. 2021;52(12):4021–32. 34719258 10.1161/STROKEAHA.120.033285 PMC8607924

