# Supplementary material for: CCL5 is essential for axonogenesis and neuronal restoration after brain injury
Source: J Biomed Sci. 2024 Sep 17;31:91. doi: 10.1186/s12929-024-01083-w (PMC11406852; doi:10.1186/s12929-024-01083-w)
Supplement: Supplementary file 8 — Supplementary Material 8. [file 12929_2024_1083_MOESM8_ESM.pdf]

Supplementary Information:

Title: CCL5 is essential for axonogenesis and neuronal restoration after brain injury

Authors:

Man-Hau Ho[1,2,†], Yih-Jeng Tsai[3,4,†], Chia-Yen Chen[2], Anastasia Yang[2,5], Thierry Burnouf[6,7,8,9,10], Yun Wang[11], Yung-Hsiao Chiang[6,12,13], Barry J Hoffer[1,2,14,15], Szu-Yi Chou[1,2,6,9,16*]

*Corresponding author: Szu-Yi Chou; E-mail(s): sichou@gmail.com, sichou@tmu.edu.tw;

†These authors contributed equally to this work.

Includes 7 Supplementary Figure legends, 7 Supplementary Figures and 1 Supplementary Table.

Supplementary Table 1: Antibodies used in the study:

Antibody	Source	Company	Car. Number	Dilution
**Primary Antibodies**				
CCL5/RANTES	Goat	R&D system	AF478	IHC: 1:100
CXCR4	Rabbit	Millipore	Ab522	WB: 1:1000
EIF2	Mouse	Abcam	Ab5369	WB: 1:1000
Phospho-EIF2 (Ser51)	Rabbit	Abcam	Ab32157	WB: 1:500
Erk	Rabbit	Cell Signaling	4695	WB: 1:2000
Phospho-Erk	Rabbit	Cell Signaling	4370	WB: 1:1000
FAK	Rabbit	Cell Signaling	3285	WB: 1:1000
Phospho-FAK	Rabbit	Cell Signaling	3283	WB: 1:1000
GAP43	Rabbit	Millipore	Ab5220	WB: 1:1000
GFAP	Rabbit	GeneTex	GTX108711	IHC: 1:1000
Iba1	Rabbit	GeneTex	GTX100042	IHC: 1:1200
NeuN	Mouse	GeneTex	GTX30773	IHC: 1:200
NRG-1	Rabbit	GeneTex	GTX101117	WB: 1:1000
Oligo-2	Rabbit	GeneTex	GTX132732	IHC: 1:500
P70S6	Rabbit	Cell Signaling	2708	WB: 1:1000
Phospho-P70S6 (Thr421/Ser424)	Rabbit	Cell Signaling	9204	WB: 1:1000
PSD95	Mouse	GeneTex	GTX634291	WB: 1:1000
Reelin	Mouse	Abcam	ab78540	IHC: 1:1000
Sema3A	Rabbit	GeneTex	GTX130671	WB: 1:1000
SMI32	Mouse	BioLegend	801701	WB: 1:1000; IHC: 1:500
Synaptophysin	Rabbit	GeneTex	GTX100865	WB: 1:2000
mTOR	Mouse	Cell Signaling	4517	WB: 1:1000
Phospho-mTOR (Ser2448)	Rabbit	Cell Signaling	5536	WB: 1:1000
Tuj-1	Mouse	GeneTex	GTX631836	ICC: 1:1000
**Secondary Antibodies**				
Phalloidin-594		Thermo Fisher Scientifics	A12381	ICC: 1:400
Alexa Fluor 488 donkey anti-mouse IgG	Donkey	Invitrogen	A21202	IHC, ICC: 1:400
Alexa Fluor 488 donkey anti-rabbit IgG	Donkey	Invitrogen	A21206	IHC, ICC: 1:400
Alexa Fluor 568 donkey anti-rabbit IgG	Donkey	Invitrogen	A10042	IHC, ICC: 1:400
donkey anti rabbit cy5	Donkey	Jackson lab	150312	ICC: 1:200
Alexa Fluor 488 donkey anti-goat IgG	Donkey	Invitrogen	A11055	IHC: 1:400
anti-Mouse IgG	Goat	Jackson lab	115-035-003	WB: 1:10,000
anti-Rabbit IgG	Goat	Jackson lab	111-035-003	WB: 1:10,000
DAPI		Sigma	D9542	IHC, ICC: 1:10,000

Supplementary Figures and Figure Legends:

Supl.Figure 1

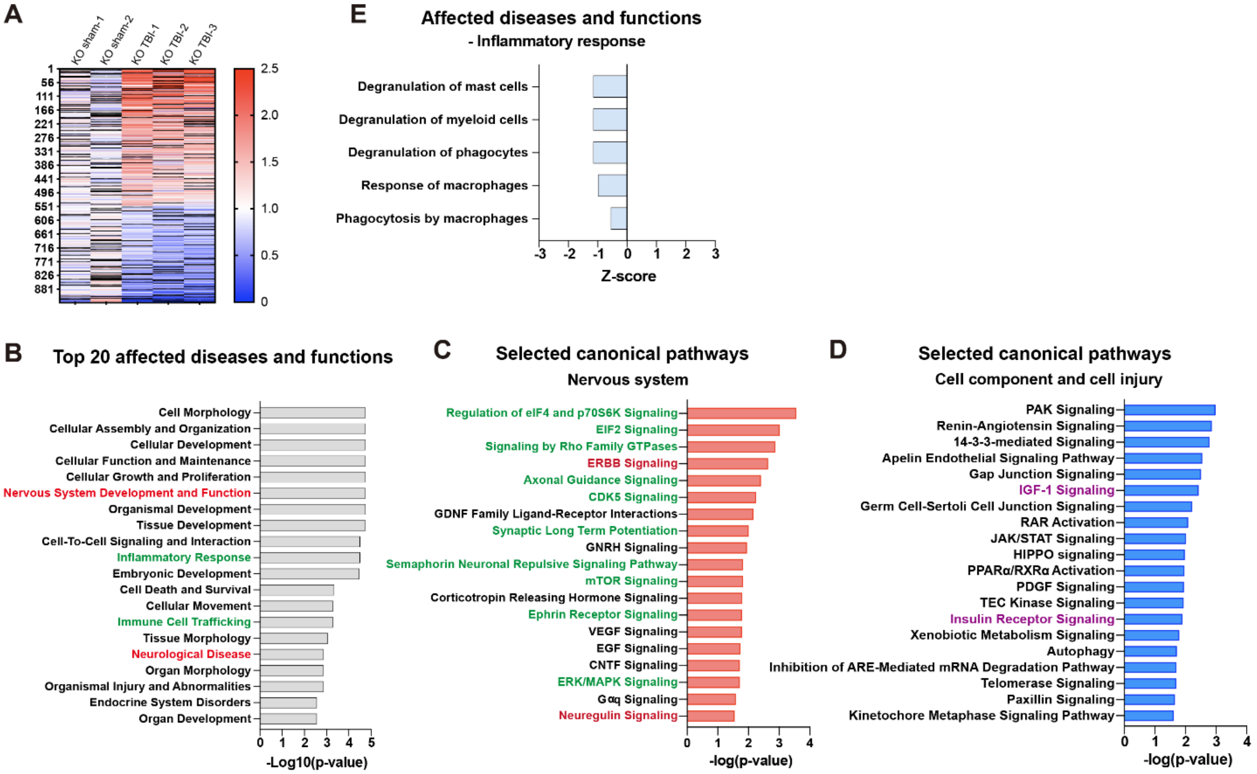

Supplementary Fig. 1: Proteomic analysis of CCL5-KO cortical tissue in sham and TBI groups. (A) The heatmap of CCL5-KO sham and TBI groups of mice. (B) The top 20 protein-affected diseases and functions in cortex tissue of CCL5-KO mice after TBI by IPA analysis. (C) The nervous system-related categories and (D) cell component and cell injury-related categories in selected canonical pathways in IPA analysis. (E) The inflammatory response-related category in affected disease and functions with IPA analysis. Z-score values indicate that functions are predicted to be inhibited (blue).

Supl. Figure 2

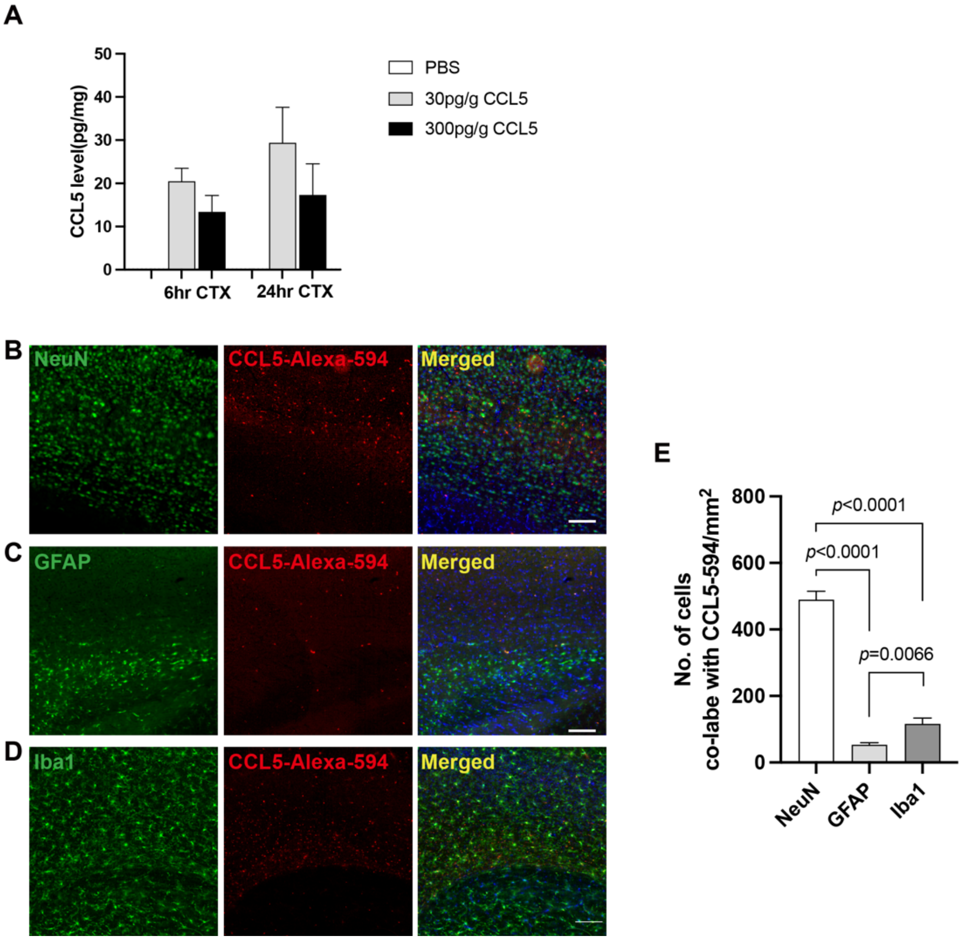

Supplementary Fig. 2: The localization of recombinant CCL5 in CCL5-KO mouse cortex. Recombinant CCL5 (rCCL5) was labeled by Alexa-594 and administered to the brains of mice through nasal administration. **(A)** The level of rCCL5 in mouse motor cortex (CTX) tissue was detected by CCL5-ELISA after 6 and 24 hrs administration. The colocalization of rCCL5-Alexa-594 (Red) to neurons **(B)**, astrocytes **(C)**, and microglia **(D)** was detected by co-labeling with NeuN (Green), GFAP (Green), and Iba1(Green). Quantification result was in **(E)** (CCL5+ in Neuron vs in GFAP, $p<0.0001$; CCL5+ in GFAP vs in Iba1, $p=0.0066$; CCL5+ in neuron vs in Iba1, $p<0.0001$). Data were analyzed by *t*-test.

Supl.Figure 3-pre vs postL5

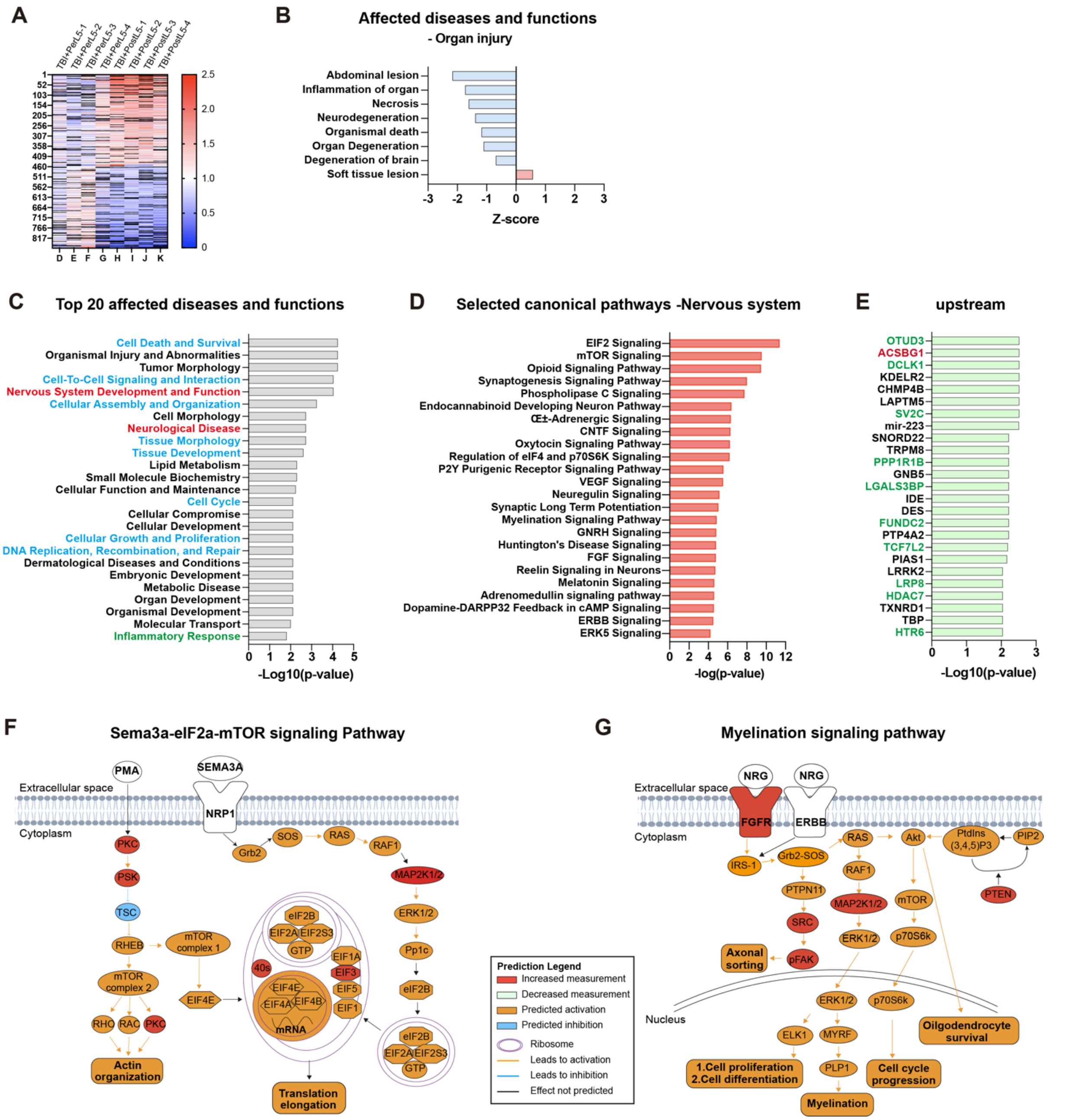

Supplementary Fig. 3: Protein pathway analysis of mice after TBI with CCL5 pretreatment and TBI with CCL5 post-treatment groups in CCL5-KO mice. (A) The heatmap of CCL5-KO TBI, TBI with Pre-treated CCL5, and TBI with Post-treated CCL5 groups of mice. (B) The organ injury category of affected diseases and function in IPA analysis. Z-score values indicate that functions are predicted to be activated (red) or inhibited (blue). (C) IPA analysis identified the Top 20 disease and function proteins. (Blue labeled cellular development and related function, Red marked nervous-related function and green labeled inflammation-related function.) (D)

Nervous system-related categories in selected canonical pathways in IPA analysis. (E) The upstream signaling molecules are identified in both PreL5 and PostL5 treatments (axonogenesis, neuritogenesis, and synaptogenesis-related are labeled green, and the myelination pathway is labeled red). (F-G) The network of DEPs is related to axon and neurite growth (F) and myelination (G).

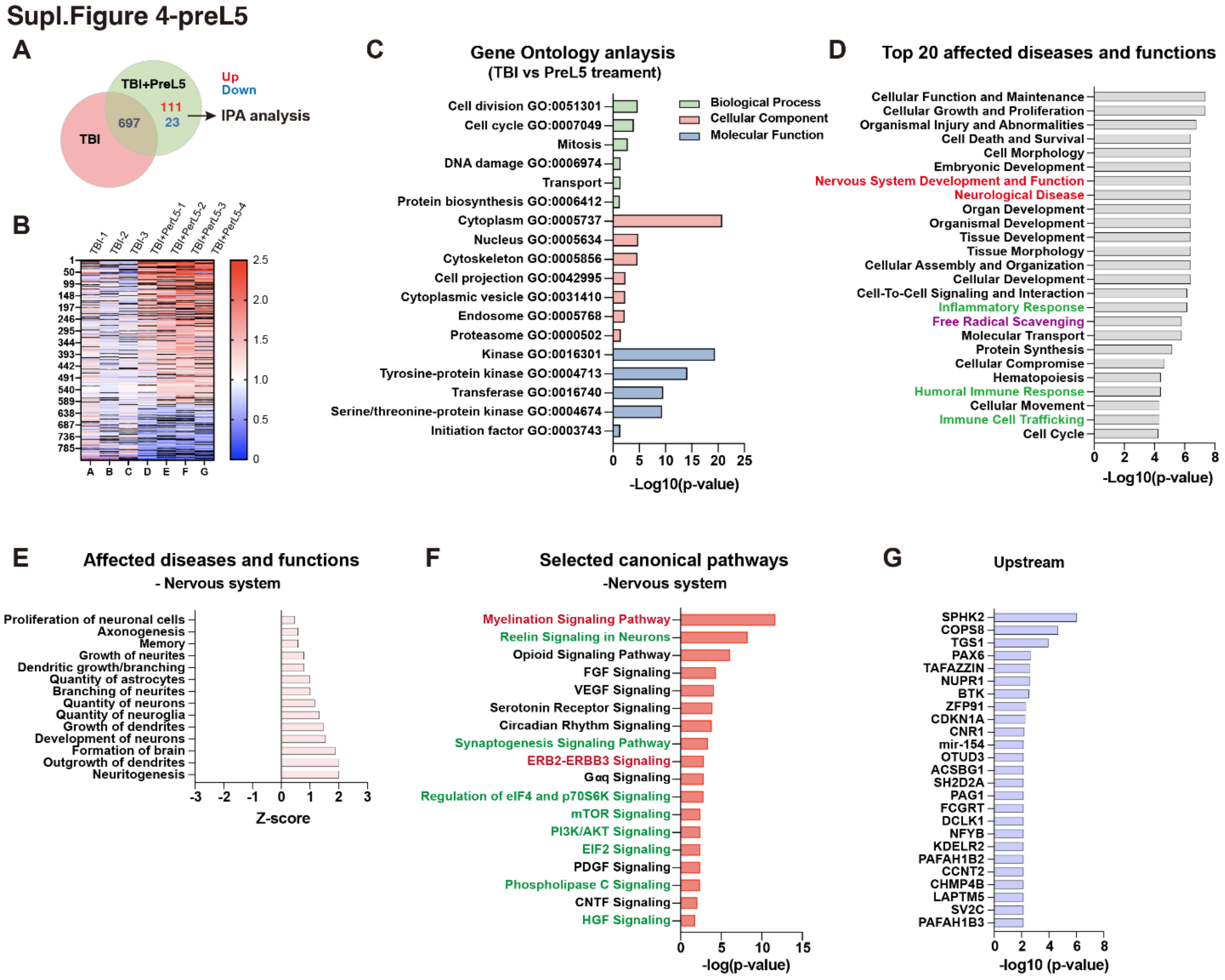

Supplementary Fig. 4: Protein pathway analysis in the TBI with CCL5 pretreatment group of CCL5-KO mice. (A) Venn diagram comparing DEPs (differentially expressed proteins) between TBI and TBI with CCL5 pretreatment (PreL5) in CCL5-KO mouse cortex. DEPs: p-value < 0.05 in comparison to TBI, respectively. Colored points represent log2 ratio > 0 upregulated genes (red) and log2 ratio < 0 downregulated genes (blue). (B) The heatmap of CCL5-KO TBI and TBI with Pre-treated CCL5 groups of mice. (C) GO enrichment analysis of 134 (111 up regulated, 23 down regulated)-identified proteins in the three GO terms are shown (Green:

biological process, Red: cellular component, Blue: molecular function). The strength of enrichment of each GO term is indicated by the Log10 p-value (X-axis). (D) IPA analysis identified the Top 20 diseases and functions. (Red labeled nervous-related function, green labeled inflammation-related function, and purple labeled oxidative-related function.) (E) The nervous system category of affected diseases and function. Z-score values indicate which functions are predicted to be activated (red). (F) Selected IPA canonical pathways in the nervous system identified significant DEPs related to axonogenesis (green) and myelination pathways (red). (G) The predicted upstream components in IPA analysis.

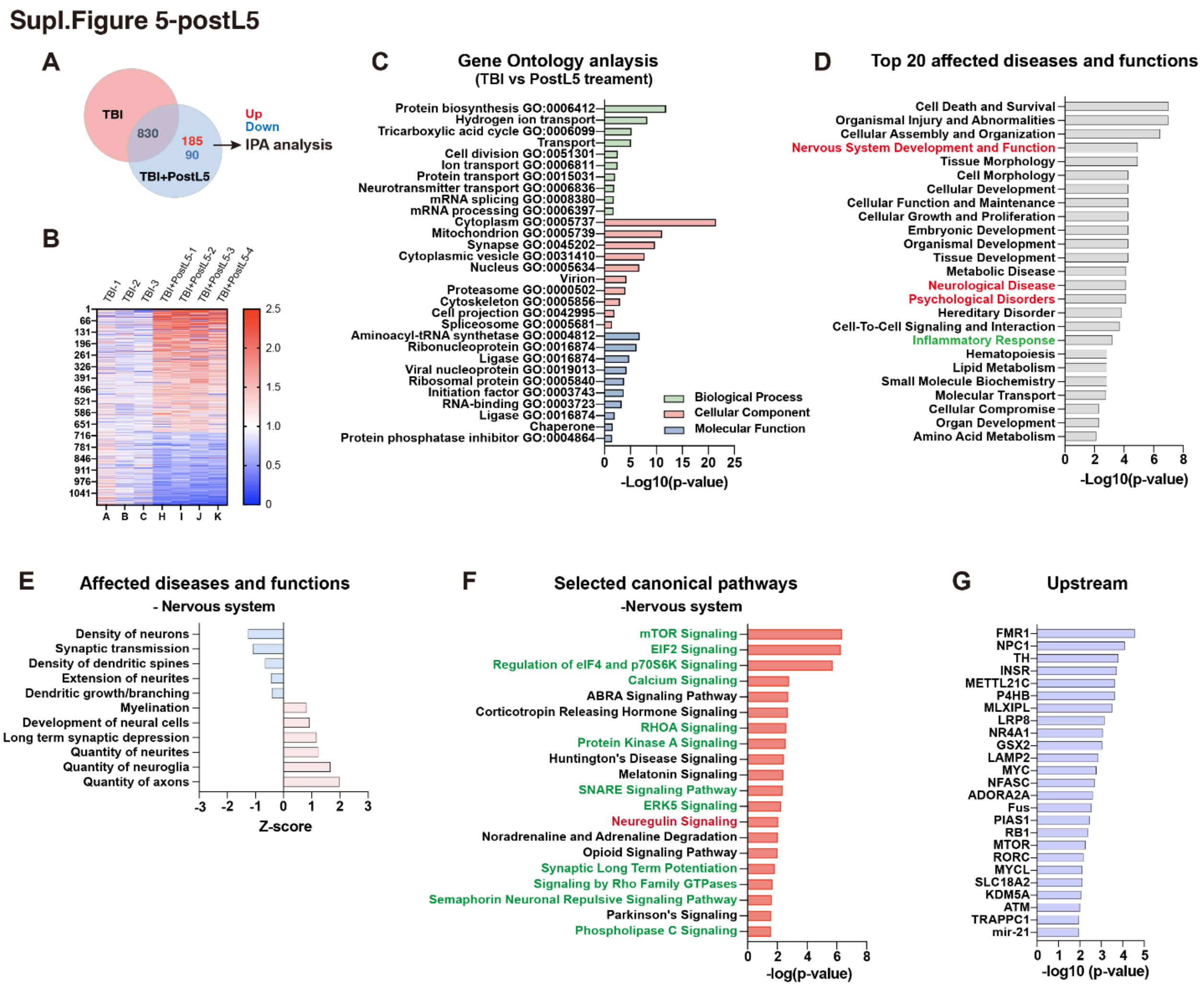

Supplementary Fig. 5: Protein pathways analysis in the TBI with CCL5 post-treatment group of CCL5-KO mice. (A) Venn diagram comparing DEPs between TBI and TBI with CCL5 post-treatment (PostL5) CCL5-KO mouse cortex. DEPs: p-value < 0.05 in comparison to TBI,

respectively. Colored points represent log2 ratio > 0 upregulated genes (red) and log2 ratio < 0 downregulated genes (blue). (B) The heatmap of CCL5-KO TBI and TBI with Post-treatment CCL5 groups of mice. (C) GO enrichment analysis of 275 (185 up regulated, 90 down regulated)-identified proteins in three GO terms are shown (Green: biological process, Red: cellular component, Blue: molecular function). The strength of enrichment of each GO term is indicated by the Log10 p-value (X-axis). (D) IPA analysis identified the Top 20 diseases and functions. (Red-labeled nervous-related function and green-labeled inflammation-related function.) (E) The nervous system category of affected diseases and function. Z-score values indicate which functions are predicted to be activated (red) or inhibited (blue). (F) Selected IPA canonical pathways in the nervous system identified significant DEPs related to axonogenesis (green) and myelination pathways (red). (G) The predicted upstream components in IPA analysis.

Supl.Figure 6

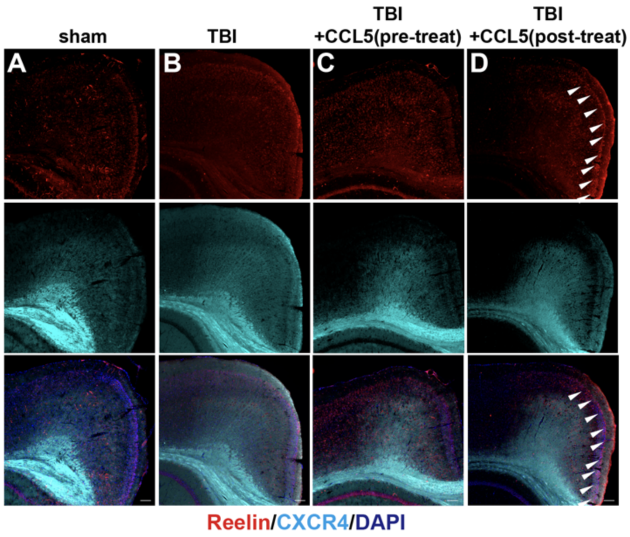

Supplementary Fig. 6: The activation of Reelin and CXCR4 in mouse cortex. The immunostaining of Reelin (red) and CXCR4 (Cyan) in different groups of mouse cortex including (A) sham, (B) TBI, (C)TBI with CCL5 pre-treatment and (D) TBI with CCL5 post-treatment groups of mice. DAPI (blue) labeled nucleus. Arrowheads point to the Reelin positive cells in (D). Scale bar = 100 µm.

Supl.Figure 7

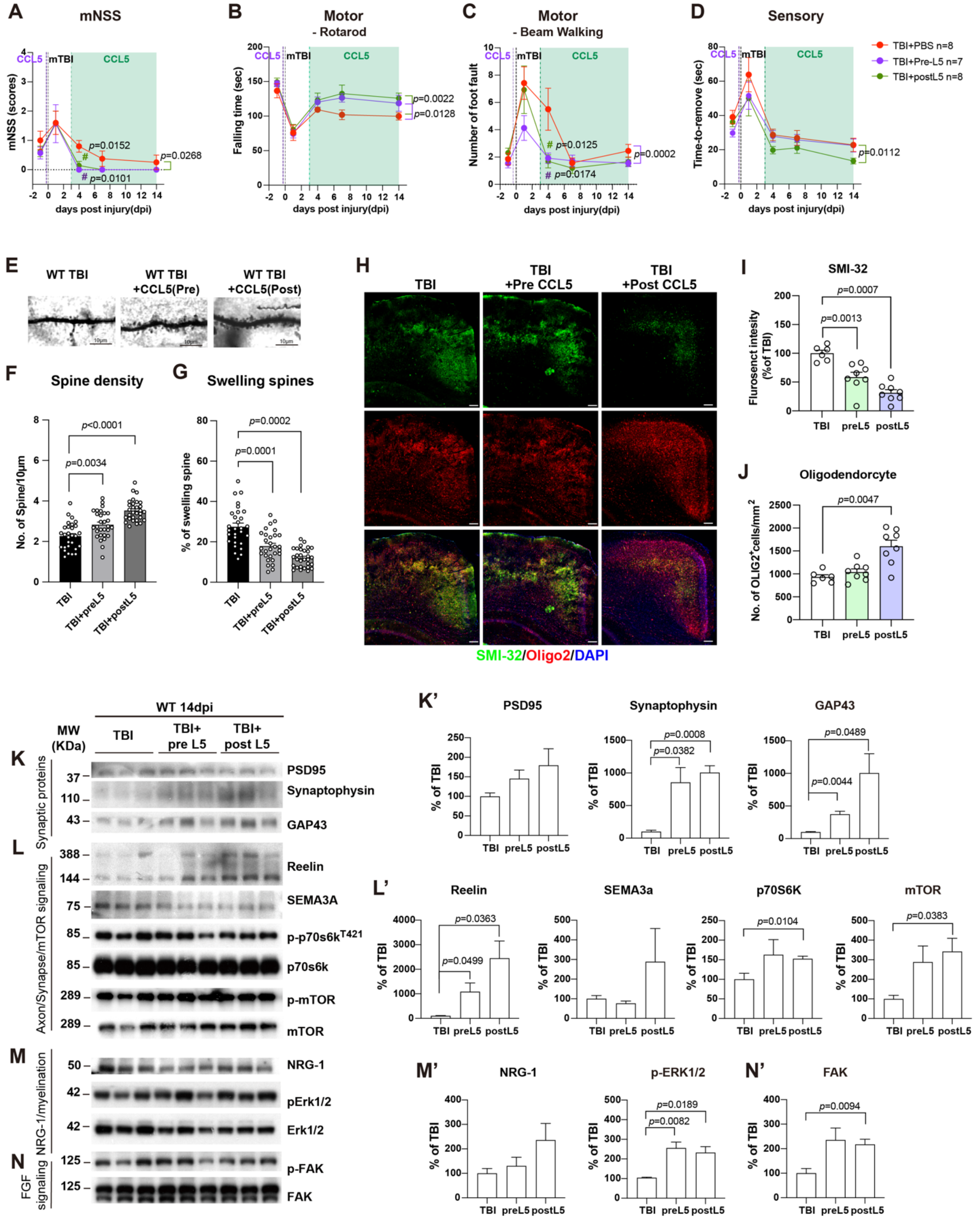

Supplementary Fig. 7: CCL5 treatment facilitated the improvement of neurological function, synapse formation and re-myelination in WT mice after mTBI. WT mice with mTBI received a

single dose of CCL5 (300 pg/g) 30 min before injury (PreL5) or received multiple treatments after 3 days of injury (PostL5) until 14 dpi (day post injury) (CCL5:30 pg/g, every 2 days until 14 dpi). (**A-D**) Black dashed line points to the time of brain injury. The Purple dashed line indicates the treatment with CCL5 before the weight drop impact; the green dashed line and green area indicate the post-treatment with CCL5. (**A**) The mNSS score, (**B-C**) Rotarod (**B**) and beam walking (**C**) was improved by i.n. CCL5 treatment, especially by multiple treatments (PostL5). (**D**) Sensory function - sticker removal test was also improved by CCL5 administration. (n=7~8 in **A-D**). (**A**: PBS vs Post-L5, *p*=0.0268; **B**: PBS vs Pre-L5, *p*=0.0022; PBS vs Post-L5, *p*=0.0128; **C**: PBS vs Pre-L5, *p*=0.0002; **D**: PBS vs Post-L5, *p*=0.0112. Data was analyzed by two-way ANOVA between groups and presented as mean± SEM. **A**: PBS vs Pre-L5 at 4dpi, p=0.0101; PBS vs Post-L5 at 4dpi, p=0.0152 by t-test. **C**: PBS vs Pre-L5 at 4dpi, *p*=0.0174; PBS vs Post-L5 at 4dpi, *p*=0.0125 by *t*-test.). **(E-G)** Golgi staining of cortical neurons in TBI, TBI with PreL5, and TBI with PostL5 groups of WT mice. (**E**) The representative images of neurites and dendritic spines in different groups of WT mice. Scale bar=10 μm. The spine density (**F**) and the number of swollen spines (**G**) were quantified in different groups of mice (slide number=10~12 in each group). (**F**: PBS vs Pre-L5, *p*=0.0034; PBS vs Post-L5, *p*<0.0001; **G**: PBS vs Pre-L5, *p*<0.0001; PBS vs Post-L5, *p*=0.0002.). (**H**) The immunostaining of unmyelinated axon - SMI-32 (green) and oligodendrocytes - oligo-2 (red) in 3 groups of WT mouse cortex. DAPI (blue) labeled the nucleus. Scale bar = 100 μm. The quantification results were in (**I**) SMI-32 and (**J**) oligodendrocytes (slide number=6~8 in each group). (**I**: PBS vs Pre-L5, *p*=0.0013; PBS vs Post-L5, *p*=0.0007; **J**: PBS vs Post-L5, *p*=0.0047.). **(K-N)** Western blot analyzed the expression of different signaling proteins in different groups of WT mouse cortex, including synaptic proteins – PSD95, synaptophysin, and GAP43 (**K, K'**); axon-related signaling proteins – Reelin, Sema3, p70S6K, and mTOR (**L, L'**); myelination-related proteins - Neuregulin, and Erk (**M, M'**); and FGF signaling - FAK phosphorylation (**N, N'**). (Quantification results were in **K'-N'**, n=3~4 in each group.) (**K'**: Synaptophysin: PBS vs Pre-L5, *p*=0.0382; PBS vs Post-L5, *p*=0.0008; GAP43: PBS vs Pre-L5, *p*=0.0044; PBS vs Post-L5, *p*=0.0489.) (**L'**: Reelin: PBS vs Pre-L5, *p*=0.0499; PBS vs Post-L5, *p*=0.0363; p70S6: PBS vs Post-L5, *p*=0.0104; mTOR: PBS vs Post-L5, *p*=0.0383.) (**M'**: ERK1/2: PBS vs Pre-L5, *p*=0.0082; PBS vs Post-L5, *p*=0.0189). (**N'**: FAK: PBS vs Post-L5, *p*=0.0004.) Data in **F-G**, **I-J**, **K'-N'** were analyzed by t-tests and presented as mean± SEM.
